# Diazotrophy affects the state transitions in unicellular nitrogen fixing cyanobacteria

**DOI:** 10.1007/s11120-026-01231-3

**Published:** 2026-07-27

**Authors:** Saverio Rana, Tatsuhiro Tsurumaki, Eva Kotabová, Radek Kaňa, Alžběta Prášilová, Takako Masuda, Ondřej Prášil

**Affiliations:** 1 Laboratory of Photosynthesis, Centre Algatech, Institute of Microbiology of the Czech Academy of Sciences, Novohradská 237, Třeboň, 379 01 Czech Republic; 2 Faculty of Science, University of South Bohemia in České Budějovice, Branišovská 1645/31a, České Budějovice, 370 05 Czech Republic; 3 Fisheries Resources Institute, Japan Fisheries Research and Education Agency, Shinha­macho, Shiogama, Miyagi Japan

**Keywords:** Cyanobacteria, State transition, Energy spillover, Phycobilisome detachment, State transition capacity

## Abstract

State transitions balance excitation energy between photosystem II and photosystem I and represent a key photoprotective mechanism in oxygenic photosynthesis. While extensively characterized in model cyanobacteria, the regulation of state transitions in diazotrophic species remains poorly understood. Here, we present a comparative physiological study of two unicellular nitrogen-fixing cyanobacteria, *Crocosphaera watsonii* WH 8501 and *Crocosphaera subtropica* ATCC 51142 under diazotrophic and non-diazotrophic conditions. Using growth analyses, whole-cell absorption spectroscopy, and low-temperature (77 K) fluorescence spectroscopy, we examined how nitrogen availability shapes photosynthetic energy distribution across species and conditions. Our results showed that state transitions are tightly linked to nitrogen metabolism in both species, but with distinct species-specific regulatory strategies. Diazotrophic cultures exhibited a higher capacity for state transitions than non-diazotrophic cultures, indicating enhanced flexibility in excitation energy redistribution. In *C. watsonii*, pronounced state transitions were observed exclusively under diazotrophic conditions, whereas non-diazotrophic cultures relied on alternative modes of excitation regulation. In contrast, *C. subtropica* displayed a more flexible response, with state transitions influenced by both nitrogen availability and light intensity. Fluorescence analyses revealed that state transitions involve a combination of excitation spillover between photosystems and dynamic coupling–decoupling of phycobilisomes. Together, these findings suggest that nitrogen fixation is associated with changes in photosynthetic energy regulation in marine diazotrophic cyanobacteria.

**Supplementary Information:**

The online version contains supplementary material available at 10.1007/s11120-026-01231-3.

## Introduction

Light harvesting is a fundamental process in photosynthetic organisms, as light is converted into biochemical energy (ATP) and reducing power (NADPH), both crucial for the fixation of the CO_2_ into organic carbon molecules. However, when the amount of energy delivered to the photosynthetic reaction centers is greater than what cellular processes can utilize, light turns into a threat rather than a benefit. In such situation, reactive oxygen species are generated in both photosystem I (PSI) and photosystem II (PSII), causing serious cellular damage. In nature, photosynthetic organisms face the energetic imbalance between PSII and PSI due to the fluctuations of light quality and quantity (Calzadilla and Kirilovsky 2020). In order to maintain an optimal distribution of excitation energy between PSII and PSI, photosynthetic organism employ a regulatory mechanism known as state transitions (STs) (Dong et al. 2009). This process aims to reallocate excitation energy from the light harvesting antenna to the two photosystems in response to changes in light quality (Bhatti et al. 2020).

STs were originally identified more than five decades ago in red alga *Porphyridium cruenium* (Murata 1969) and green alga *Chlorella pyrenoidosa* (Bonaventura and Myers 1969). In these studies, due to changes in fluorescence yield based on the light used, two functional states were characterized: State I, in which PSII is preferentially excited, and State II, in which PSI is preferentially excited. In past decades mostly plants and green algae were used as model organisms for STs studies (Williams and Allen 1987; Forsberg and Allen 2001; Allen 2002). Researchers attributed the change in the fluorescence spectra of PSI and PSII of these organisms to the partial migration to the Light-Harvesting Complex II (LHCII), the main membrane chlorophyll-carotenoid binding PSII antenna. Selective illumination shifts the redox balance of the plastoquinone (PQ) pool, involving cytochrome b_6_f, driving PQ toward a more reduced or oxidized state. STs act as a regulatory response to counter this imbalance by reallocating excitation energy between the two photosystems. The two states also differ in their fluorescence patterns: State II is characterized by a higher PSI/PSII fluorescence ratio, whereas in State I this ratio decreases.

In cyanobacteria, light harvesting is mediated by the phycobilisome (PBS), a large (3–18 MDa) peripheral protein complex (Sui 2021) attached to the cytoplasmic side of the thylakoid membrane and composed of an ordered network of bilin-containing pigment-protein complexes (Domínguez-Martín et al. 2022). Numerous studies have shown that STs in cyanobacteria are driven by changes in light quality that alter the redox state of the PQ and likely involve dynamic changes in the phycobilisome functional organization (PBS) (Rakhimberdieva et al. 2001; Kaňa et al. 2012; Tamary et al. 2012; Zhao et al. 2014; Chukhutsina et al. 2015). However, despite extensive investigation, there is still no consensus on the molecular mechanisms underlying STs in cyanobacteria (Kirilovsky et al. 2014; Calzadilla and Kirilovsky 2020).

Since the initial discovery of STs in cyanobacteria, several factors have been suggested to play a role in the overall mechanistic model of STs controlling energy redistribution between photosystems. The first factor, the mobile PBS antenna, suggests that PBSs can migrate fast laterally along the thylakoid membrane and physically associate with either PSII (state I) or PSI (state II) (Mullineaux et al. 1997; Rakhimberdieva et al. 2001; Zhao et al. 2014). FRAP microscopy data supported the high degree of PBS mobility required for the mechanism, on the other hand the super-resolution microscopy rather disprove the fast PBS mobility on thylakoid membrane surface (Mullineaux et al. 1997; Kaňa et al. 2023). Therefore, long-distance PBS migration is now considered unlikely to be the dominant mechanism (Calzadilla and Kirilovsky 2020). The second factor involved in STs is represented by the energy spillover between photosystems. It proposes that the optimal excitation energy (re-)distribution from PSII to PSI proceeds via PSII core antenna protein CP47 through direct chlorophyll-chlorophyll interactions (McConnell et al. 2002). The third factor is the PBS detachment. It proposes that the transition from state I to II involves also the partial or complete functional or physical uncoupling of PBS from PSII, thereby reducing excitation delivery to PSII (Tamary et al. 2012; Kaňa et al. 2012). Finally, as the fourth factor, the effect of fluorescence quenching in PSII has been also proposed (Bhatti et al. 2020, 2021). Time-resolved fluorescence studies have shown that state II is characterized by increased excited-state quenching of PSII chlorophylls, which reduces PSII fluorescence yield independently of PBS movement or spillover (Bhatti et al. 2020). Together, these findings indicate that cyanobacterial STs arise from a combination of PBS mobility, energetic decoupling, spillover, and intrinsic PSII quenching, rather than a single universal mechanism (Calzadilla and Kirilovsky 2020).

Most of the existing research has focused on model cyanobacterial strains such as *Synechocystis* sp. PCC 6803 (Chen et al. 2015; Zhao et al. 2015) and *Synechococcus elongatus* PCC 7942 (Campbell et al. 1996; Kaňa et al. 2012; Ranjbar Choubeh et al. 2018). In contrast, much less is known about how state transitions are regulated in diazotrophic cyanobacteria in response to nitrogen availability. Diazotrophs strongly adjust their metabolism depending on nitrogen status because nitrogen fixation is highly energy demanding and closely linked to photosynthesis. Therefore, changes in how energy is distributed between the two photosystems may be part of their acclimation to nitrogen conditions.

In unicellular diazotrophs *C. subtropica* (formerly *Cyanothece)*, previous studies have shown changes in fluorescence and photosystem behavior linked to the daily cycle, including PBS-related transitions, but only under fully diazotrophic growth (Meunier et al. 1997). In another diazotroph, *Plectonema boryanum*, temporal separation of photosynthesis and nitrogen fixation is associated with state-dependent redistribution of excitation energy between PSI and PSII, suggesting a link between nitrogen metabolism and light-harvesting regulation (Misra and Mahajan 2000). The contrasting ecological niches of *C. watsonii* and *C. subtropica* likely require different strategies of light and energy regulation, making these species an informative comparative system for studying state transitions in diazotrophic cyanobacteria. However, it is still unclear how state transitions respond to nitrogen availability in *Crocosphaera* and whether they are similar between different species.

In this study, we investigated STs under diazotrophic and non-diazotrophic conditions in two species of unicellular nitrogen-fixing cyanobacteria: the globally ecologically relevant *Crocosphaera watsonii* WH 8501 and the laboratory model *Crocosphaera subtropica* (formerly *Cyanothece*) ATCC 51142, hereafter *C. watsonii* and *C. subtropica*, respectively. This study provides a comparative physiological characterization of how the two species respond to nitrogen availability, with focus on photosynthetic energy redistribution between photosystems. Our results demonstrate that nitrogen availability plays a key role in regulating STs and reveal species-specific differences in photosynthetic acclimation in these unicellular diazotrophic cyanobacteria.

## Materials and methods

### Strains and growth conditions

Stock cultures of *C. watsonii* and *C. subtropica* were maintained in N-free YBCII medium (Chen et al. 1996) at 28 °C in glass flasks, shaken at 100 rpm under constant white light (100 µmol photons m^− 2^ s^− 1^) using a 12:12 h light-dark (12L:12D) cycle. At the beginning of each experiment, stock cultures were centrifuged at 6000 × g for 10 min, and the resulting cell pellets were resuspended directly in the 100 mL culture tubes containing fresh YBCII medium. Cultures were prepared either without added nitrogen (diazotrophic condition) or supplemented with 1 mM NH₄Cl or 17.6 mM NaNO₃ for *C. watsonii* and *C. subtropica*, respectively (non-diazotrophic conditions). The tubes were then placed in a Multi-Cultivator MC 1000-OD device (Photon Systems Instruments, Drasov, Czech Republic) with sinusoidal light 12L:12D cycle, peaking at 50 (LL) or 200 (HL) µmol photons m^− 2^ s^− 1^. All treatments were run in triplicate, and cultures were bubbled with ambient air (400 ppm CO_2_). Cells were maintained below an optical density 720 nm (OD_720_) of 0.5 to avoid light limitation and were diluted to an OD_720_ of 0.25 whenever this threshold was reached, typically every two days. Dilutions were performed manually by resuspending the cells in fresh YBCII medium. This was repeated to reach steady state over a period of at least 14 days.

### Absorption spectra

Absorption spectra were measured using Unicam UV 550 spectrometer (ThermoFisher, UK), equipped with an integrating sphere. A 10 mL aliquot of the culture was filtered through a Pragopor 5 nitrocellulose membrane filter (pore size 0.6 μm; Pragopor, Czech Republic). The filter was subsequently placed in an integrating sphere, and spectral measurements were acquired with a 1 nm wavelength increment over the 400–800 nm range. Blank spectra were measured using a clean membrane filter and subtracted from the raw absorption spectra. The baseline corrected absorption spectra were then normalized to the 680 nm peak.

### Low temperature fluorescence spectroscopy

Fluorescence emission spectra were measured at 77 K using a SM-9000 spectrophotometer (Photon Systems Instruments, Drasov, Czech Republic), either with 450 nm (chlorophyll excitation) or 590 nm excitation light (phycobilisome excitation). The cells were collected at 6 h into the light period (6 L). An aliquot of 1 mL culture sample was immediately filtered through Whatman™ GF/F glass fibers filter (Cytiva, United Kingdom), mounted in a copper sample holder, placed in a liquid nitrogen containing optical Dewar flask with a transparent finger. Additionally, a separate 1 mL aliquot of the sample was dark-adapted for 10 min prior to fluorescence measurements at 77 K. Blank spectra were acquired using a moistened filter and subtracted from the corresponding raw spectra. All fluorescence measurements were performed in triplicate and averaged. Low-temperature (77 K) fluorescence emission spectra were subsequently deconvoluted by PeakFit^®^ (Systat Softwear Inc., San Jose, California, USA). The resulting deconvoluted peak areas were used to calculate the relative ratios of photosynthetic complexes (e.g., PSI/PSII, PBS/PSII). These ratios were then used to determine the STs capacity, calculated as the difference between PSI/PSII in dark- and light-adapted cells.

### PBS orthologous gene search

Orthologous proteins of PBS were searched by KEGG BLASTP (https://www.genome.jp/tools/blast/) with the restriction of organism database “cwa cyt syn synu synk syw syx” (Kanehisa et al. 2024). Both *Crocosphaera* and *Synechocystis* PBS genes were used as queries reciprocally. Protein IDs of OCP were referred from (Bao et al. 2017; Sheppard et al. 2025). Functional categories of each protein were further confirmed in Cyanorak (Garczarek et al. 2020). Since *C. watsonii* WH 8501 contains phycoerythrin (PE), functional categories of PE were further confirmed by protein BLAST in Cyanorak as followed (Garczarek et al. 2020). PE sequences of C. watsonii WH 8501 were used as queries, then the function was assigned based on the Cluster Number (CK_xxxxxxxx) of the BLAST top hit. 

### Isolation of thylakoid membranes and Clear Native (CN)-PAGE

*C. watsonii* and *C. subtropica* cells were grown under diazotrophic or non-diazotrophic conditions. Cultures were maintained at an irradiance of 50 µmol photons m^− 2^ s^− 1^ under a 12:12 h light-dark cycle. Samples were collected after 6 h of illumination by centrifugation. Thylakoid membrane preparation and CN-PAGE in a 4–14% polyacrylamide gradient gel were performed as described by (Komenda et al. 2011). Solubilized thylakoid membranes corresponding to 5 µg of chlorophyll for *C. watsonii* and 3 µg of Chl for *C. subtoropica* were loaded per lane.

### Statistics

We have used standard statistical methods to calculate means and standard deviations. Statistical comparisons of absorption spectra in Fig. 2 were performed using Welch’s t-test based on means, standard deviations, and replicate number, without assuming equal variance between conditions. For each absorbance peak, absolute differences and percentage changes between -N and + N were calculated. To account for multiple comparisons across peaks and conditions, p-values were adjusted using the Benjamini-Hochberg false discovery rate correction.

## Results

### Growth

**Fig. 1:**
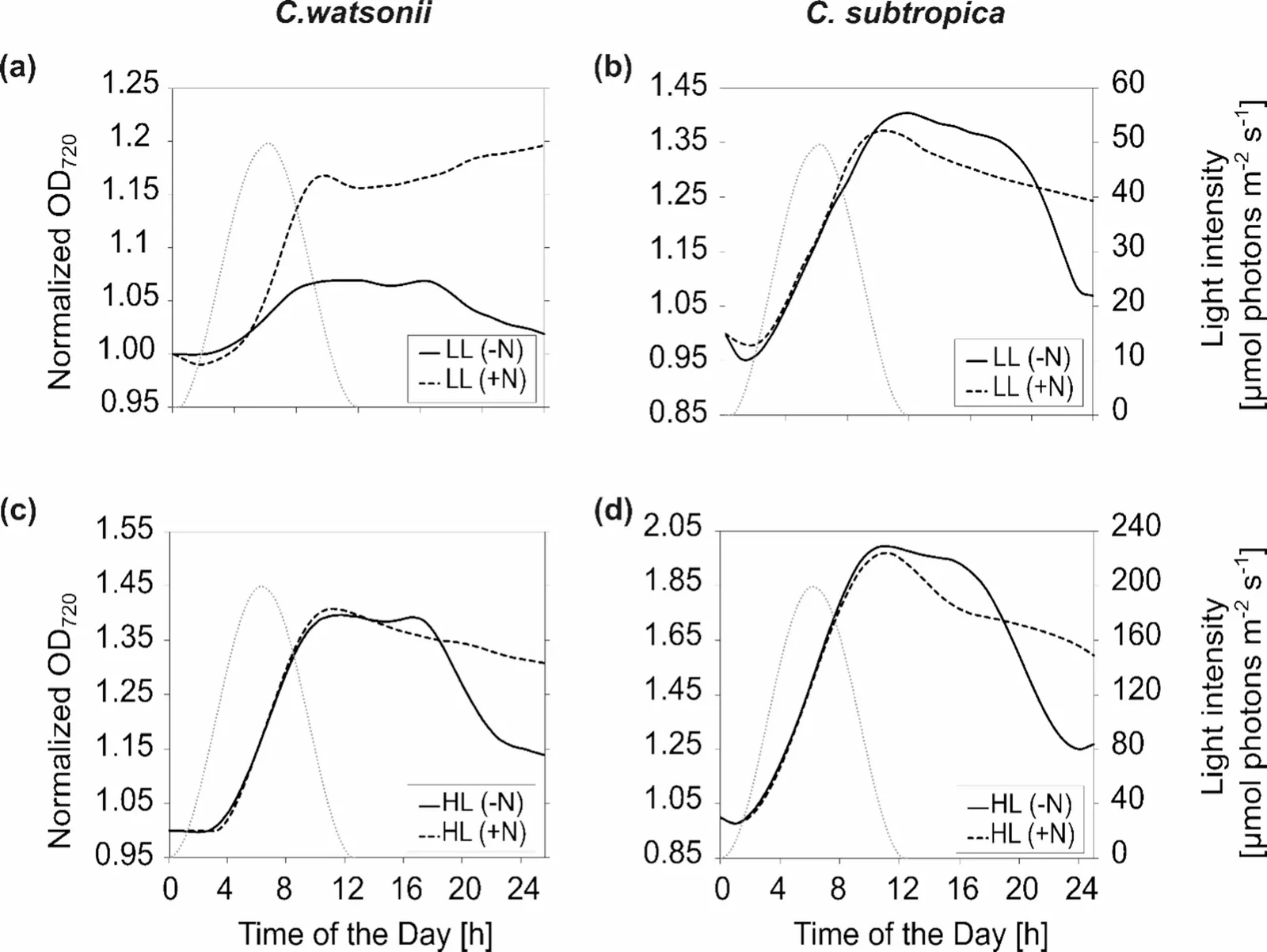
Growth dynamics of *Crocosphaera* cultures under diazotrophic and non-diazotrophic conditions. Daily changes in OD_720_ of *C. watsonii* (**a, c**) and *C. subtropica* (**b, d**) cultures, where OD_720_ was normalized to OD_720_ values at 0 L (beginning of light period). OD_720_ was monitored under a 12L:12D light-dark cycle at low light (LL, 50 µmol photons m^-2^ s^-1^) and high light (HL, 50 µmol photons m^-2^ s^-1^). Cultures were grown under diazotrophic (-N; solid line) and non-diazotrophic (+ N; dashed line) condition. Grey dotted line indicates the relative light intensity. Both species exhibited diel growth patterns, with increases in OD_720_ during the light period and declines during the dark phase particularly under diazotrophic conditions. Data represent averages from *n* = 3 biological replicates. SD are not shown because of graphical reasons

Growth dynamics was assessed by monitoring daily changes in optical density at 720 nm (OD_720_). A clear diurnal pattern in OD_720_ was observed with growth occurring predominantly during the light phase (between 4 and 12 h of day) in both *C*. *watsonii* and in *C. subtropica*. We evaluated the effects of light intensity and DIN on growth during this period (Fig. 1). Increased irradiance enhanced growth in both strains, as HL conditions resulted in higher OD_720_ compared to LL conditions. On the contrary, no effect of DIN was detected in *C. watsonii* under HL conditions (Fig. 1c), nor in *C. subtropica* under either LL or HL conditions (Fig. 1b, d). However, an increase in OD_720_ associated with nitrogen availability was observed in *C. watsonii* under LL conditions (Fig. 1a). Except for this response, daily growth in both strains under diazotrophic conditions was predominantly driven by light intensity rather than nitrogen availability.

Following the transition from light to dark, a pronounced decline in OD_720_ was observed in diazotrophic cultures, whereas non-diazotrophic cultures continued to increase or showed a modest decline of OD_720_, thereby producing distinct diel responses between diazotrophic and non-diazotrophic cultures (Fig. 1). Under LL conditions, diazotrophic *C. watsonii* exhibited a clear decline in OD_720_, while non-diazotrophic cultures showed continued growth (Fig. 1a), likely attributable to anaplerotic reactions. In contrast, under HL conditions, *C. watsonii* exhibited in the dark a decline in OD_720_ under diazotrophic conditions that was approximately 4-fold greater than under non-diazotrophic conditions. *C. subtropica* instead showed a greater decrease in OD_720_ in the dark under diazotrophic compared to non-diazotrophic conditions. The rates of decline under diazotrophy were approximately 3-fold higher under LL and 2-fold higher under HL compared to controls. These results indicate that the decreases in OD_720_ at night may reflect consumption of polysaccharide granules formed in the light period by carbon fixation and used for nitrogen fixation (Masuda et al. 2018, 2022).

### Pigment content

**Fig. 2:**
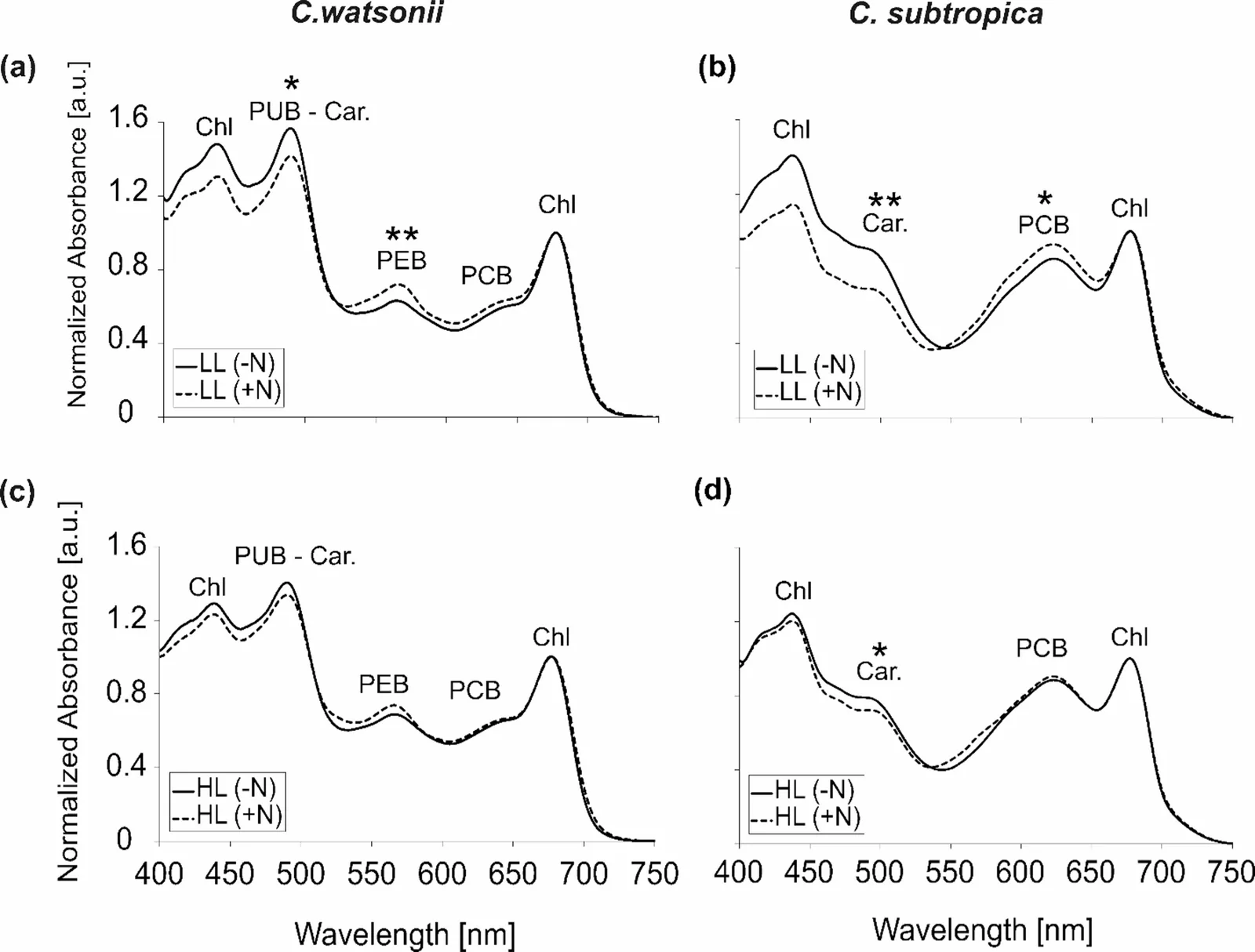
Nitrogen-dependent changes in cellular pigment composition. Absorption spectra of *C. watsonii* (**a, c**) and *C. subtropica* (**b, d**) cultures. Spectra were collected at 6 h into the light period (6 L) under low light (LL, 50 µmol photons m^-2^ s^-1^) and high light (HL, 200 µmol photons m^-2^ s^-1^) and normalized to the Chl maxima of the Qy band (678 nm). Cultures were grown under a 12L:12D light-dark cycle under diazotrophic (-N; solid line) and non-diazotrophic (+ N; dashed line) conditions. Nitrogen availability altered pigment composition, particularly in the phycobiliproteins absorption region. Nitrogen-dependent significant differences in the peak maxima are indicated by asterisks, ** indicates significance after Benjamini-Hochberg correction. Data represent averages from *n* = 3 biological replicates. SD are not shown because of graphical reasons

Nitrogen-dependent changes in the pigmentation of the cells were inferred from the whole cell absorption spectra measured at 6 h into the light period (6 L). In non-diazotrophic *C. watsonii* cultures, a statistically significant increase in absorption at 560 nm (phycoerythrobilin, PEB) was observed under LL (Fig. 2a). In contrast, under diazotrophic conditions, there was a significant increase in absorption in the Soret band of chlorophyll a (Chla) (430–450 nm) and in the region around 470–500 nm, associated either with phycourobilin (PUB) and carotenoids (Car) that could not be resolved.

Like the observation in *C. watsonii*, we detected a significantly higher absorption in the Soret band of Chla and carotenoids under LL diazotrophic conditions in *C.subtropica* when compared to the non-diazotrophic cultures (Fig. 2b, d). Additionally, PCB levels were higher in LL non-diazotrophic cultures compared to their diazotrophic counterpart highlighting a distinctive spectral response linked to nitrogen availability. Moreover, relative carotenoid-associated absorption (470–500 nm) was higher under diazotrophic conditions also under HLintensities.

### Low temperature fluorescence spectroscopy

The Low-temperature (77 K) fluorescence emission spectra were used to study PSI/PSII ratio, PBS decoupling and progress of STs under diazotrophic and non-diazotrophic conditions.

**Fig. 3:**
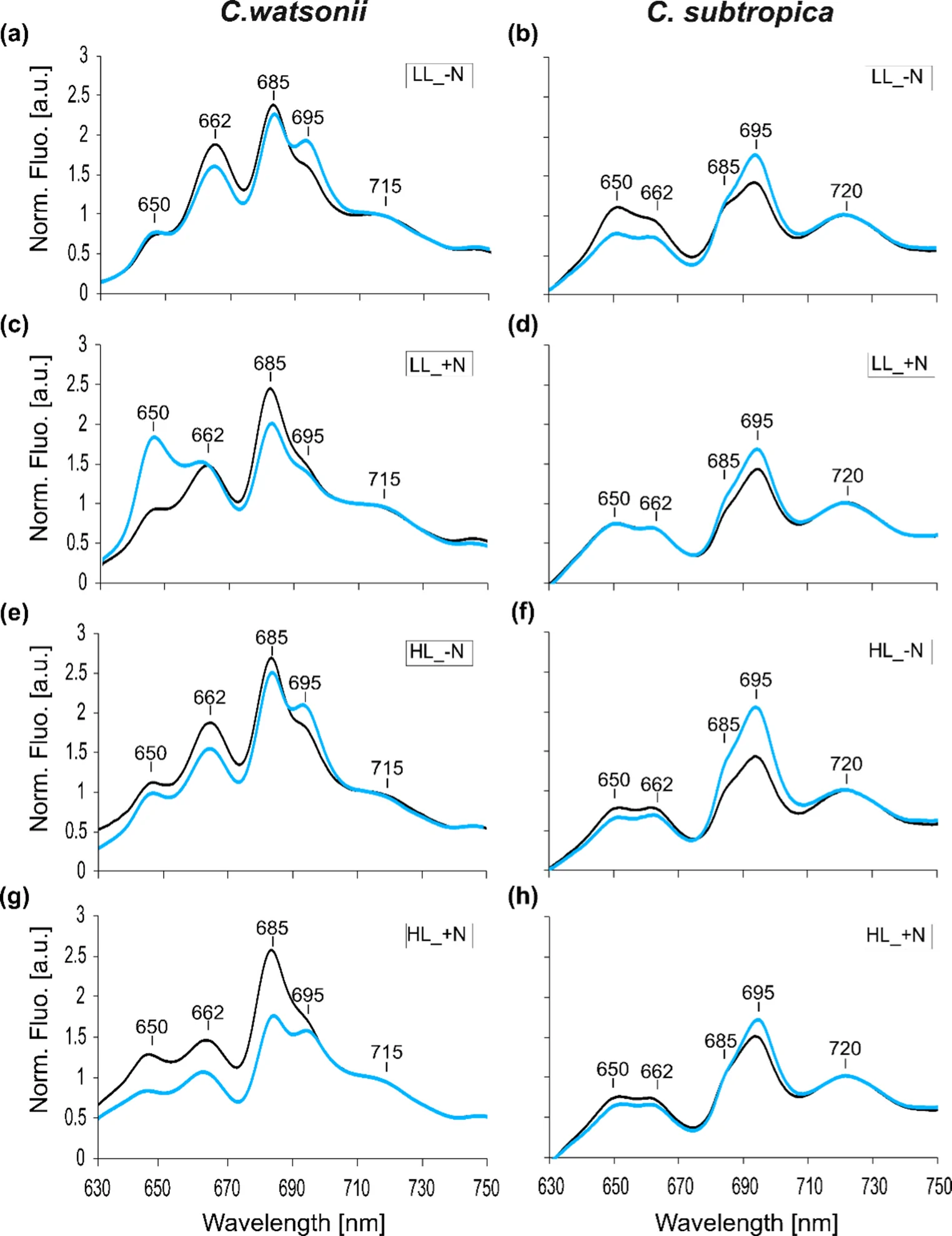
Low temperature (77 K) fluorescence emission spectra under PBS excitation at 590 nm of *C*. *watsonii* (**a, c, e, g**) and *C*. *subtropica* (**b, d, f, h**) measured under diazotrophic (-N) and non-diazotrophic (+N) condition, at low (LL, 50 µmol photons m^-2^ s^-1^) and high light (HL, 200 µmol photons m^-2^ s^-1^). Spectra were normalized to the F715 emission peak for *C. watsonii* and the F720 peak for *C. subtropica*. Blue lines represent samples measured 6 h into the light period (6 L), whereas black lines represent samples collected at 6 L and dark-acclimated for 10 min prior to measurement. Differences between light- and dark-adapted spectra indicate changes in excitation energy distribution between PBS and the photosystems. Data represent averages from *n*=3 biological replicates. SD are not shown because of graphical reasons

The 77 K emission spectra for Chl 450 nm (Fig. S1) and excitation to PBS (590 nm – see Fig. 3) revealed five major peaks (Murata et al. 1966; Masuda et al. 2018; Calzadilla and Kirilovsky 2020) with different intensities for Chl-PBS excitation (Fig. S1, Fig. 3): (1) F650 band was dominant during excitation into PBS as it corresponds to emission of phycocyanin; (2) F662 band was also dominant during excitation into PBS as it is related to allophycocyanin; (3) F685 band, was dominant under both excitations as it is related to the PBS terminal emitter with contribution from CP43 emission; (4) F695 band was dominant under both excitations corresponding to emission from CP47 and reaction center II and (5) F715 and F720 bands linked to PSI in *C. watsonii* and *C. subtropica*, respectively. The latter bands (F695 and F715-720) obtained under Chl excitation (Fig. S1) reflected fluorescence originating from PSII and PSI, respectively, allowing calculation of PSI/PSII ratio. On the other hand, spectra recorded under PBS excitation (Fig. 3) were used to quantify PBS decoupling by calculating the ratio of PBS fluorescence (F650 + F662) to the PSII-related band F695 (Fig. 5), which serves as an indicator of the energy transfer efficiency from PBS to PSII.

**Fig. 4:**
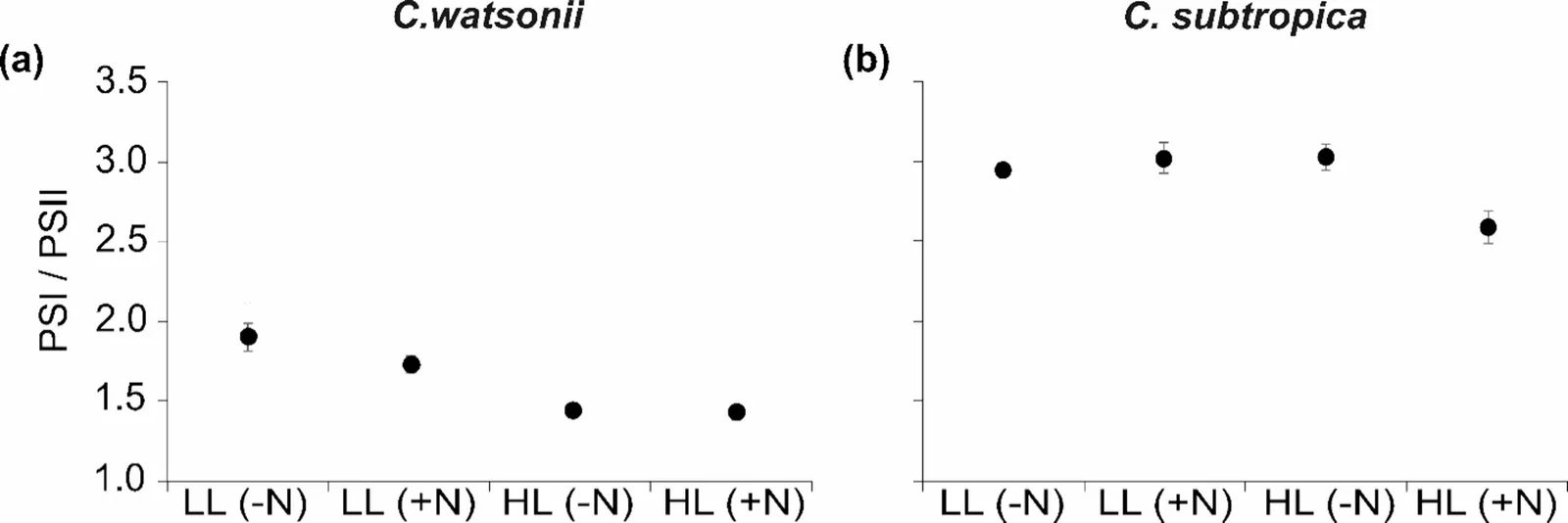
Comparison of PSI/PSII fluorescence ratios between *C. watsonii* and *C. subtropica*. Changes in PSI-to-PSII ratio (excitation at 450 nm) in *C. watsonii* (**a**) and *C. subtropica* (**b**). The PSI-to-PSII ratio was measured under diazotrophic (-N) and non-diazotrophic (+ N) conditions, as well as under low (LL, 50 µmol photons m^-2^ s^-1^) and high light (HL, 200 µmol photons m^-2^ s^-1^) treatments. Black circles represent samples collected at 6 L and dark-acclimated for 10 min prior to measurement. Error bars represent standard deviations (*n* = 3 biological replicates). The PSI/PSII fluorescence ratio differed between the two species, indicating species-specific differences in photosystem stoichiometry

Using Chl excitation (450 nm), the emission bands in the regions 695 –715 nm for *C. watsonii* and 695 –720 nm for *C. subtropica* (Fig. S1), measured after dark acclimation of the cells, were deconvoluted into individual bands corresponding to PSII and PSI (F695, F715 or F720). We then used the deconvoluted band areas to calculate the parameter “PSI/PSII fluorescence ratio”. The data (Fig. 4) revealed that *C. watsonii* displayed only slightly higher PSI/PSII ratio under LL compared to HL. In contrast, in *C. subtropica*, the PSI/PSII ratio was similar under LL and HL conditions, except for HL non-diazotrophic cells, which showed a lower ratio compared to the other conditions. More importantly, when we compare changes in the PSI/PSII ratio between the two species, we noticed that in *C. watsonii* the PSI/PSII ratio was significantly lower (approximately 1.8-fold) in comparison to *C. subtropica* across the conditions tested. Nevertheless, this pattern suggests relatively stronger PSII-associated fluorescence contributions in *C. watsonii* under the conditions examined.

**Fig. 5:**
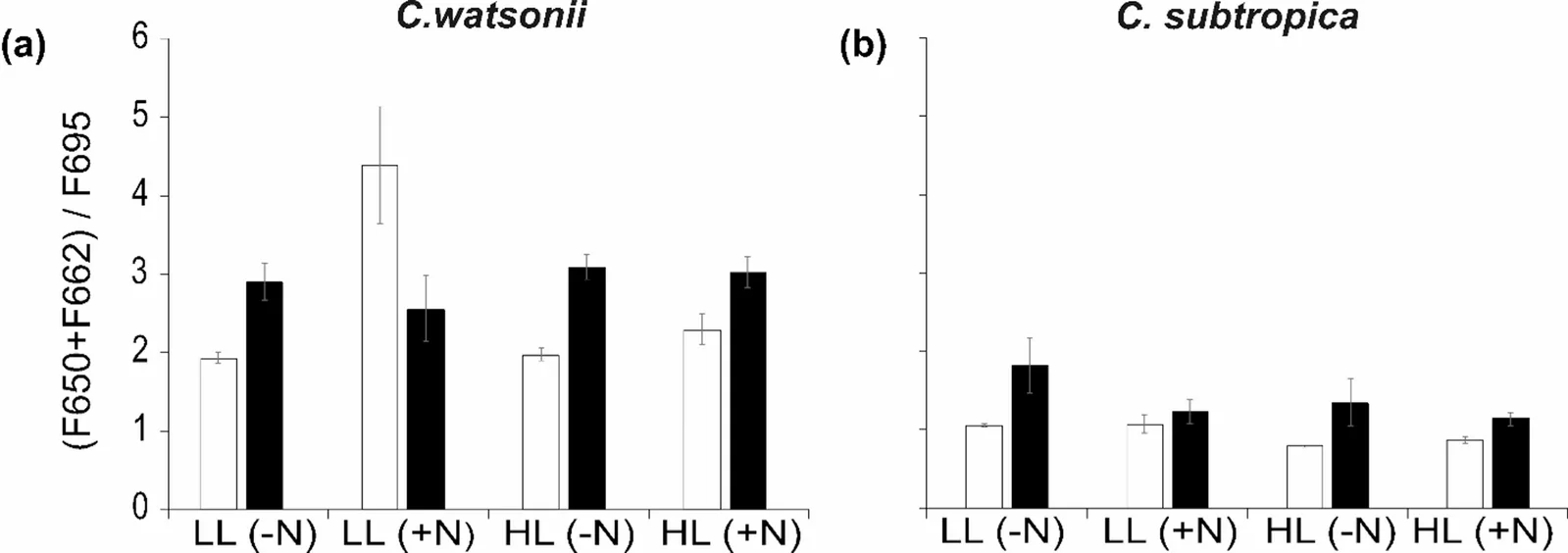
PBS-PSII coupling inferred from fluorescence emission ratios. Phycobilisome (PBS; F650 + F662) to photosystem II (PSII; F695) fluorescence ratios derived from deconvoluted peak areas of 77 K fluorescence emission spectra (excitation at 590 nm) in *C*. *watsonii* (**a**) and *C*. *subtropica* (**b**). The PBS/PSII fluorescence ratio was used as an indicator of the coupling efficiency between PBS and PSII. An increase in the PBS/PSII ratio indicates reduced excitation energy transfer from PBS to PSII consistent with partial PBS decoupling. Measurements were performed under diazotrophic (-N) and non-diazotrophic (+ N) conditions, at low light (LL, 50 µmol photons m^-2^ s^-1^) and high light (HL, 200 µmol photons m^-2^ s^-1^). Open bars represent samples measured 6 h into the light period (6 L), whereas filled bars represent samples collected at 6 L and dark-acclimated for 10 min prior to measurement. Error bars represent standard deviations (*n* = 3 biological replicates). Higher PBS/PSII ratios in dark-incubated samples indicate reduced functional coupling between PBS and PSII, consistent with dynamic regulation of excitation energy transfer

We further studied efficiency of energy transfer from PBS to photosystems based on the low-temperature (77 K) fluorescence spectra (excitation to PBS at 590 nm - see Fig. 3). In light-acclimated cells, PSII emission (F695) was higher than in dark-acclimated cells across most treatments. In contrast, non-diazotrophic *C. watsonii* exhibited the opposite pattern, with lower PSII emission in light-acclimated cells relative to dark-acclimated cells (Fig. 3c, g). By deconvoluting the emission peaks corresponding to PBS (F650-662) and PSII (F695), we assessed the coupling-decoupling dynamics of PBS from PSII during the light-to-dark transition. The PBS/PSII fluorescence ratio (F650 + F662)/F695 was used as an indicator of the functional coupling efficiency between PBS and PSII (Fig. 5). As observed in Fig. 3, except for LL non-diazotrophic *C. watsonii*, both strains displayed an increase in PBS/PSII ratios following 10 min of dark incubation (Fig. 5).

**Fig. 6:**
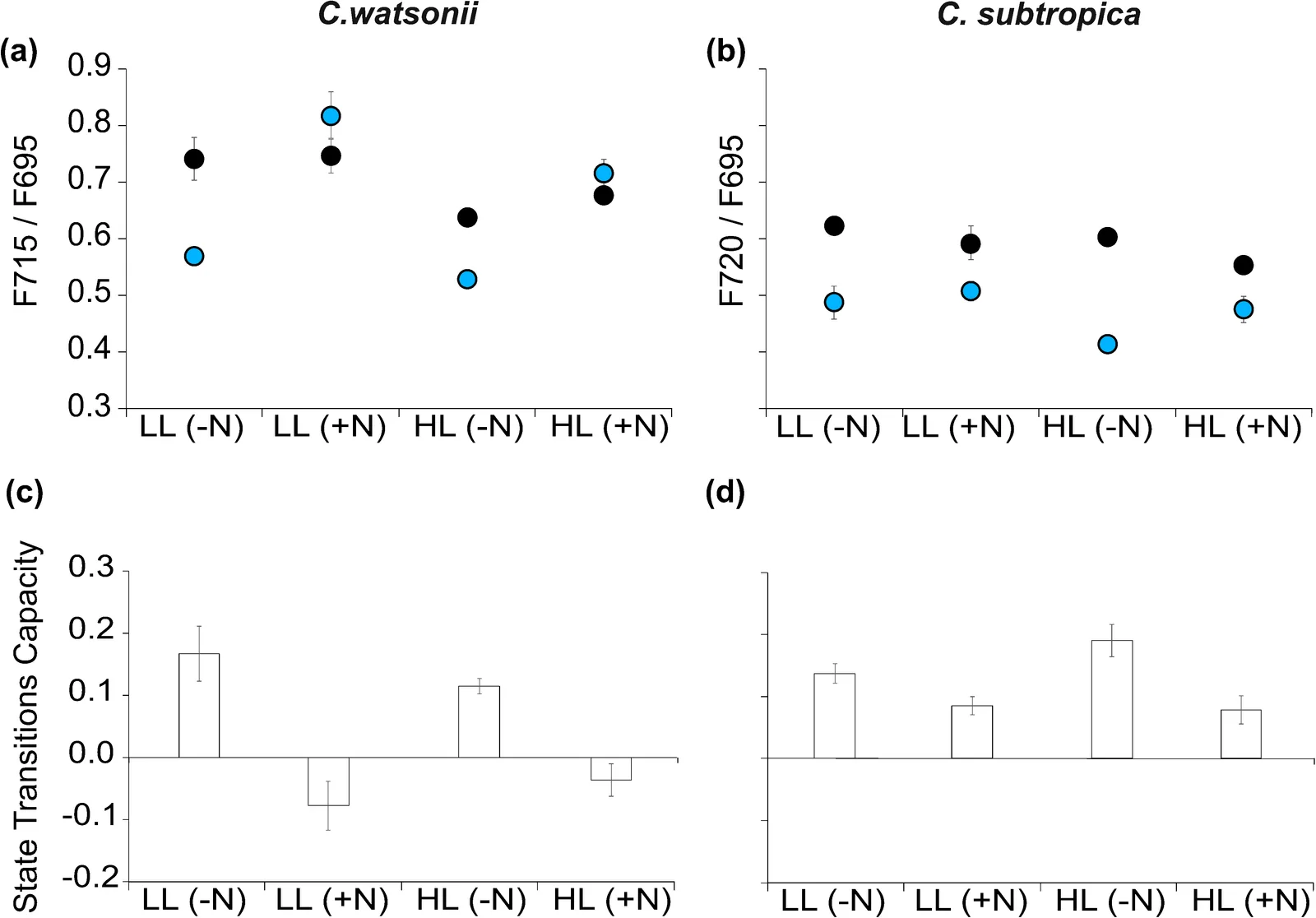
Fluorescence-based analysis of state transition dynamics. (a and b) Fluorescence emission ratios derived from deconvoluted peak areas of 77 K fluorescence spectra (excitation at 590 nm): F715/F695 for *C. watsonii* (**a**) and F720/F695 for *C. subtropica*, determined under diazotrophic (-N) and non-diazotrophic (+ N) conditions at low light (LL, 50 µmol photons m^-2^ s^-1^) and high light (HL, 200 µmol photons m^-2^ s^-1^). Blue circle represents samples measured 6 h into the light period (6 L), whereas black lines represent samples collected at 6 L and dark-acclimated for 10 min prior to measurement. (c and d) State transition capacity was calculated as (F715/F695)_dark_ – (F715/F695)_light_ for *C. watsonii* (**c**) and as (F720/F695)_dark_ – (F720/F695)_light_ for *C. subtropica* (**d**), shown as white bars. Error bars represent standard deviations (*n* = 3 biological replicates). The F715 or F720/F695 fluorescence ratio reflects the relative distribution of excitation energy between the two photosystems

Further, by deconvoluting the peak areas corresponding to PSII (F695) and PSI (F715-720) from the 77 K fluorescence spectra obtained under PBS excitation (Fig. 3), we quantified the extent of STs between light- and dark-adapted samples (Fig. 6a, b). In *C. watsonii*, the F715/F695 ratio was significantly higher (1.36-1.42-fold) in non-diazotrophic cultures than in diazotrophic culture. In contrast, *C. subtropica* exhibited much smaller differences, the F720/F695 ratio was only 1.15-fold higher under HL non-diazotrophic cultures, while no significant change was observed under LL conditions.

Using the (F715-720)/F695 ratio in light- and dark-acclimated cells, we calculated the STs capacity (Fig. 6c, d) as a descriptor of the cellular ability to rapidly shift between state I (in the light) and state II (in the dark) following changes in light conditions. This “state transition capacity” parameter turned out to be significantly higher under diazotrophic conditions. In *C. watsonii*, the highest STs capacity was observed in the LL diazotrophic condition, followed by the HL diazotrophic cultures (Fig. 6c). In contrast, both non-diazotrophic conditions exhibited smaller and negative trends, indicating that these cells did not transition to state II during dark incubation, but rather remained, or became further stabilized, in state I. In *C. subtropica* (Fig. 6d), the highest STs capacity was observed in HL followed by the LL diazotrophic cultures. In contrast, both non-diazotrophic conditions exhibited the lowest and nearly identical transition capacities, though in this case the trend remained positive.

## Discussion

In this study, we investigated the relationship between STs and diazotrophy in the unicellular nitrogen-fixing cyanobacteria *Crocosphaera watsonii* WH 8501 and *Crocosphaera subtropica* ATCC 51142 Our results showed that STs in *Crocosphaera* genus are linked to nitrogen metabolism (Fig. 3, Fig. S1). The strong coupling between STs and diazotrophy suggests that these transitions regulate excitation energy to meet the high energetic demands of N_2_ fixation, ensuring generation of sufficient carbon reserves for night-time respiration (Bandyopadhyay et al. 2013; Shimakawa et al. 2021).

Suppression of diazotrophy was associated with an overall reduction in STs capacity (Fig. 6c, d). The ability to rapidly shift between state I and state II, a mechanism that optimizes photosynthetic efficiency, was substantially reduced under non-diazotrophic conditions. This effect was most pronounced in *C. watsonii* (Fig. 6c), which showed a strong decline in STs capacity when supplied with DIN. In contrast, *C. subtropica* maintained STs capacity even under non-diazotrophic growth, although at lower levels than during diazotrophic conditions (Fig. 6d).

These patterns suggest that non-diazotrophic cells require less dynamic redistribution of excitation energy between PSII and PSI, consistent with the overall reduced metabolic demand when N₂ fixation is suppressed. Under these conditions, the need for strict photochemical energy balancing across the diel cycle is likely lower, as dependence on stored carbon for night-time metabolism decreases (Rabouille et al. 2021). The comparatively higher STs capacity retained by non-diazotrophic *C. subtropica* relative to *C. watsonii* could be related to species-specific regulatory differences, possibly linked to adaptation to their native environments. *C. watsonii*, which typically inhabits stable oligotrophic open-ocean regions with relatively constant light and nutrient availability, appears to favor tighter diazotrophy-dependent regulation of STs. In contrast, *C. subtropica*, adapted to more variable coastal environments, exhibits more flexible regulation of STs.

Our data provide insight into the possible mechanisms associated with STs in the genus *Crocosphaera.* Four main processes have been proposed to contribute to STs in cyanobacteria: (1) phycobilisome (PBS) movement, (2) energy spillover between photosystems, (3) PBS decoupling, and (4) quenching at the PSII reaction center [see review (Kirilovsky et al. 2014; Calzadilla and Kirilovsky 2020)]. Based on low-temperature fluorescence spectra, STs in *Crocosphaera* show patterns consistent with contribution from spillover (Fig. S1) and PBS detachment (Fig. 3), together with PBS-associated quenching (Fig. 5), reflecting multiple overlapping processes that may contribute to differences in excitation energy redistribution between PBS and the photosystems.

Low-temperature emission spectra for Chl excitation (Fig. S1) further suggest that the energy transfer mechanism under diazotrophic conditions, across all light intensities, could involve spillover, a process that also appears to operate in non-diazotrophic *C. subtropica.* In this cyanobacterium, the higher PSII emission at 695 nm observed in light-acclimated cells decreased after dark acclimation, indicating redistribution of excitation energy toward PSI during State II. In contrast, non-diazotrophic *C. watsonii* did not show substantial differences between light- and dark-acclimated cells, suggesting that spillover does not significantly contribute to STs under these conditions.

Our results indicate that PBS decoupling could be pronounced in *C. watsonii* grown under LL, non-diazotrophic conditions (Figs. 3c and 5a). In these cultures, partial decoupling of PBS rods (Fig. 3c) may contribute to a photoprotective function. Moreover, an increase in the PBS/PSII ratio in the light (Fig. 5a) is consistent with partial PBS decoupling from PSII (F695), with reversible re-coupling in the dark. This was the only condition in which PBS decoupling was observed. Under all other tested conditions, variations in the PBS/PSII ratio derived from 77 K spectra (590 nm excitation) could reflect PBS-based non-photochemical quenching rather than physical decoupling. Accordingly, dark incubation consistently resulted in increased PBS fluorescence emission in both *Crocosphaera* genus (Figs. 3 and 5). However, these observations are consistent with relaxation of PBS-related fluorescence quenching and may involve mechanisms associated with OCP-dependent regulation (see the following paragraph). Overall, our results suggest that PBS decoupling may represent an additional regulatory strategy in non-diazotrophic *C. watsonii*. However, because the observed fluorescence changes could also be explained by PBS-related quenching processes or other regulatory mechanisms, the contribution of PBS decoupling requires further investigation. In contrast, under the other examined conditions, variations in the PBS/PSII ratio are more likely associated with PBS-related fluorescence quenching. Nevertheless, alternative regulatory mechanisms cannot be excluded.

**Fig. 7:**
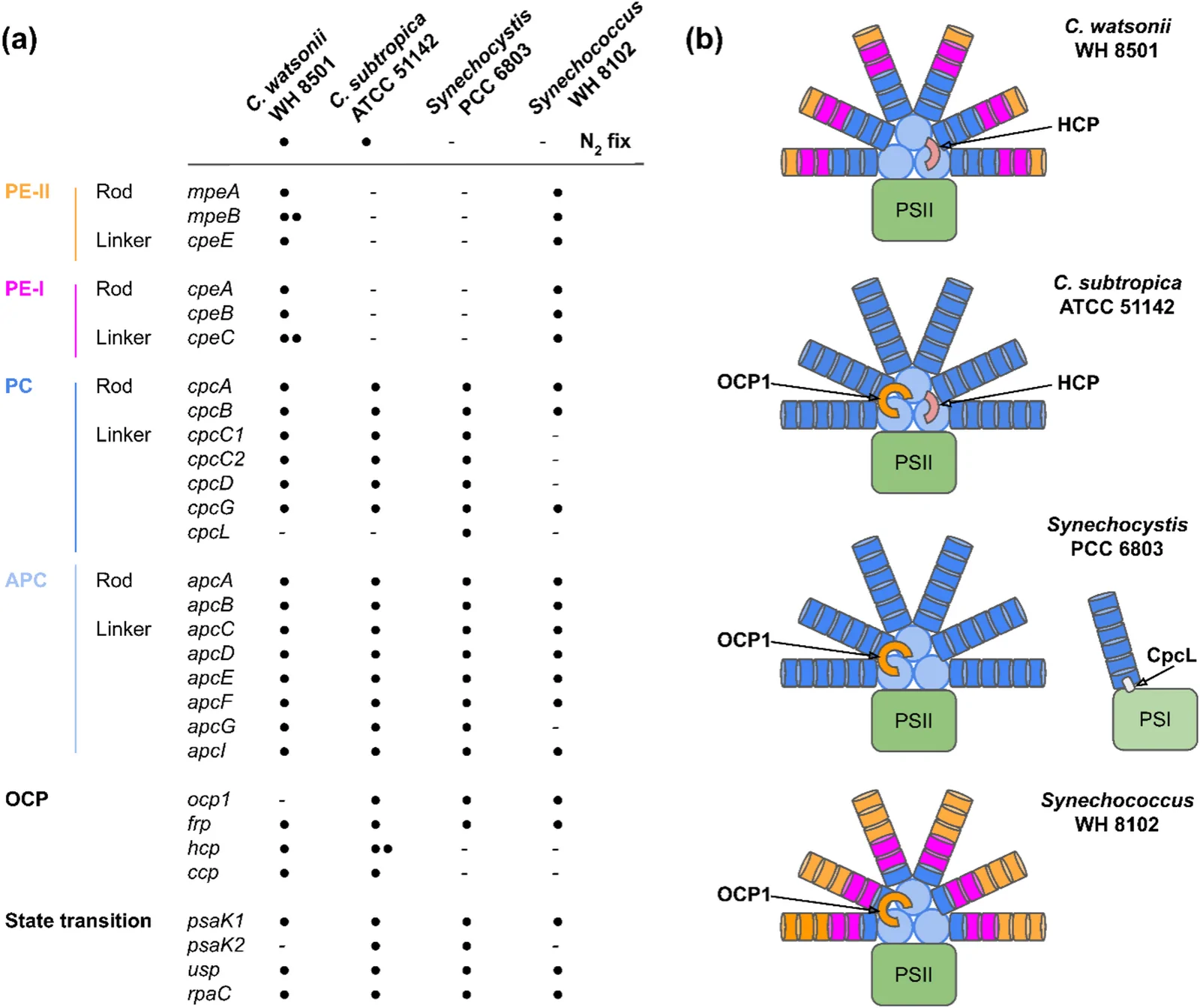
Comparison of PBS genes composition and reconstituted phycobilisome models. (**a**) Plot of orthologous genes related to PBS, quenching and state transition. “●” and “-” shows the presence or absence of nitrogen fixation (N_2_ fix) and corresponding genes, respectively. The number of “●” indicates the number of homologs in the organisms. PE-II, PE-I, PC and APC corresponds to phycoerythrin II, phycoerythrin I, phycocyanin and allophycocyanin, respectively. (**b**) Reconstituted PBS model based on gene composition. The comparison highlights the absence of canonical OCP in *C. watsonii* and the presence of alternative carotenoid-binding proteins (HCP, CCP). Arrows indicate OCP1, HCP and CpcL, respectively. PE-II (orange), PE-I (magenta), PC (blue) and APC (light blue) are colored according to the reference (Six et al. 2007). *Synechocystis* PCC 6803 and *Synechococcus* WH 8102 are shown as model organisms for comparison. The differences in PBS-related gene composition suggest species-specific phycobilisome architectures and distinct strategies for regulating excitation energy transfer

To further explore the genetic basis of this molecular mechanism of STs or PBS quenching, we used available literature to compare PBS-related genes in *C. watsonii* and *C. subtropica* including also *Synechocystis* sp. PCC 6803 and *Synechococcus* sp. WH 8102 as model organisms. *C. subtropica* possesses a PBS similar to *Synechocystis*, whereas *C. watsonii* possesses a chimeric PBS combining features of *Synechocystis* and *Synechococcus*, particularly in PE and linkers of PC (Fig. 7). Interestingly, the PBS composition of *C. watsonii* is unique from all known marine *Synechococcus* (Six et al. 2007; Grébert et al. 2022). Since it is well studied that marine *Synechococcus* species differentiate PBS genes to achieve their niche regarding to the depth in ocean (Grébert et al. 2022), the unique PBS of *C. watsonii* implies not only evolutionary origin but also ecological distribution. However, the relative distribution between *Synechococcus* and *C. watsonii* in ocean is not reported (Sato et al. 2010). It’s worthy of note that both *Crocosphaera* lack *cpcL*, which is responsible for PSI specific rod PBS (Kubushiro et al. 2025), suggesting that intact PBS structure are the primary mediators of energy transfer. Regulatory factors USP and RpaC, well characterized in *Synechocystis* (Joshua and Mullineaux 2005; Fukunaga et al. 2024), are also present in both *Crocosphaera* species. Therefore, we propose that both *Crocosphaera* species may share a mechanism of STs similar to that of *Synechocystis*, although the detailed molecular basis of this process remains under debate even in *Synechocystis* (Mullineaux and Emlyn-Jones 2004; Krumova et al. 2010).

OCP is known as the component of the OCP-dependent non-photochemical quenching (NPQ), which dissipates excess excitation energy from PBS (Kerfeld et al. 2003; Kirilovsky and Kerfeld 2012). Bioinformatic analyses have shown that *C. watsonii* lacks the canonical OCP but encodes alternative carotenoid-binding proteins, helical carotenoid protein (HCP) and C-terminal domain-like carotenoid protein (CCP), whereas *C. subtropica* retains orthologs as one *ocp*, two *hcp* and one *ccp* (Fig. 7) (Bao et al. 2017; Muzzopappa et al. 2017). Compared with *Synechocystis*, the gene composition of *C. subtropica* implies its higher ability of quenching excess excitation energy.

Despite these genetic differences, PBS-associated OCP-type quenching may represent a potential photoprotective mechanism within the *Crocosphaera* genus. However, in *C. watsonii*, quenching was not evident under low-light, non-diazotrophic growth. Under these conditions, PBS decoupling was observed, suggesting differences in the energy dissipation responses between non-diazotrophic and diazotrophic growth. Although OCP-type quenching may be broadly conserved, the present observations raise the possibility that *C. watsonii* exhibits distinct photophysiological responses under specific environmental and metabolic conditions, potentially associated with changes in organization of photosynthetic complexes. CN-PAGE analyses of photosystem complexes (Fig. S2) support this interpretation: *C. watsonii* exhibited differences in the PSI-trimer abundance, with diazotrophic cultures showing higher PSI-trimer levels than non-diazotrophic cultures. In contrast, *C. subtropica* showed changes primarily in PSII organization, with diazotrophic cells exhibiting higher PSII-dimer and PSII-monomer complexes compared to non-diazotrophic conditions. These findings highlight the species-specific tuning of energy distribution and photoprotection in *Crocosphaera*, linking PBS compositions, STs, and diazotrophic metabolism.

In conclusion, our results demonstrate that STs in *Crocosphaera* are tightly regulated by diazotrophy and differ markedly between the two studied species under nitrogen-replete conditions. These species-specific regulatory patterns likely reflect ecological adaptation to their distinct native habitats, emphasising the role of environmental niches in shaping photosynthetic energy regulation. Spectroscopic analyses further revealed possible differences in PBS-PSII coupling and STs capacity, indicating distinct strategies for regulating excitation energy transfer. These functional differences may be associated with variations in PBS-related gene composition, suggesting a role of PBS architecture in determining species-specific photophysiological response. These findings provide new insights into the interplay between nitrogen fixation and photosynthetic energy regulation in marine diazotrophic cyanobacteria.

## Supplementary Information

Below is the link to the electronic supplementary material.

Supplementary Material 1

Supplementary Material 2

Supplementary Material 3

## Data Availability

The data that support the findings of this study are available on request from the corresponding author. The data are not publicly available due to privacy or ethical restrictions.
