# Supplementary material for: Diazotrophy affects the state transitions in unicellular nitrogen fixing cyanobacteria
Source: Photosynth Res. 2026 Jul 27;164(4):43. doi: 10.1007/s11120-026-01231-3 (PMC13408156; doi:10.1007/s11120-026-01231-3)
Supplement: Supplementary file 1 — Supplementary Material 1 [file 11120_2026_1231_MOESM1_ESM.pdf]

Figure S2

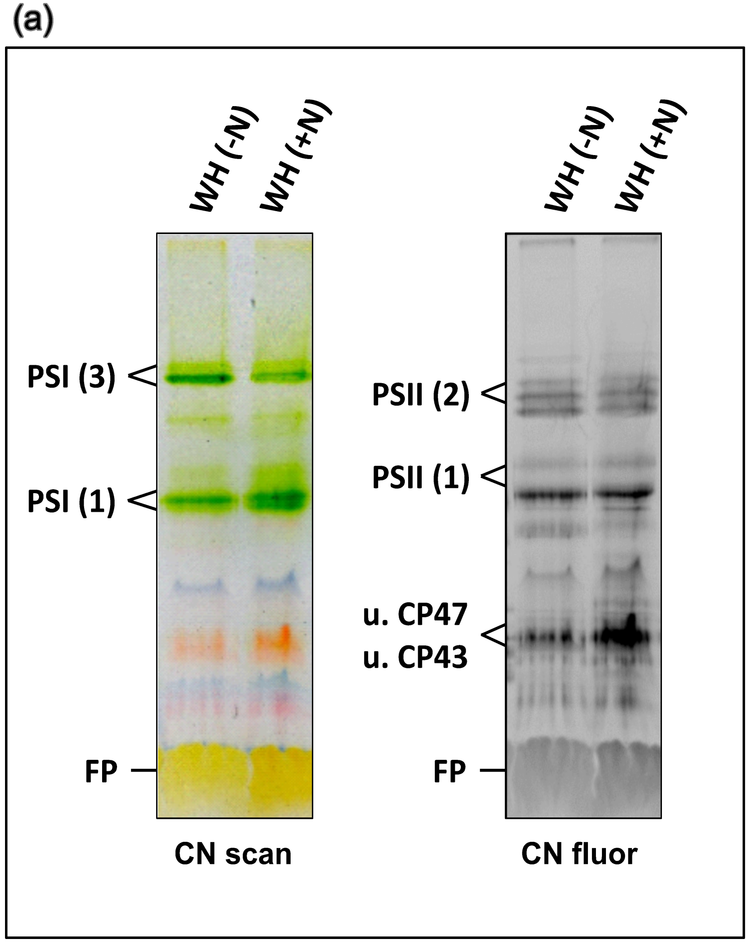

CN scan

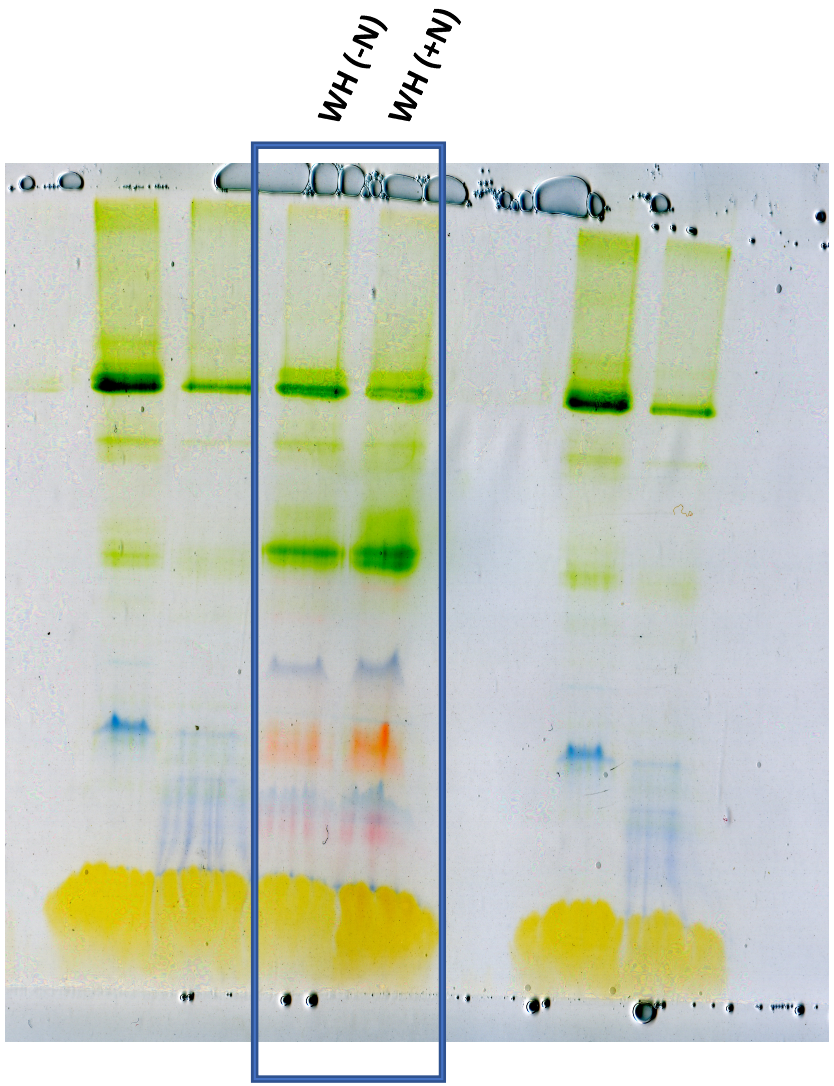

CN fluor

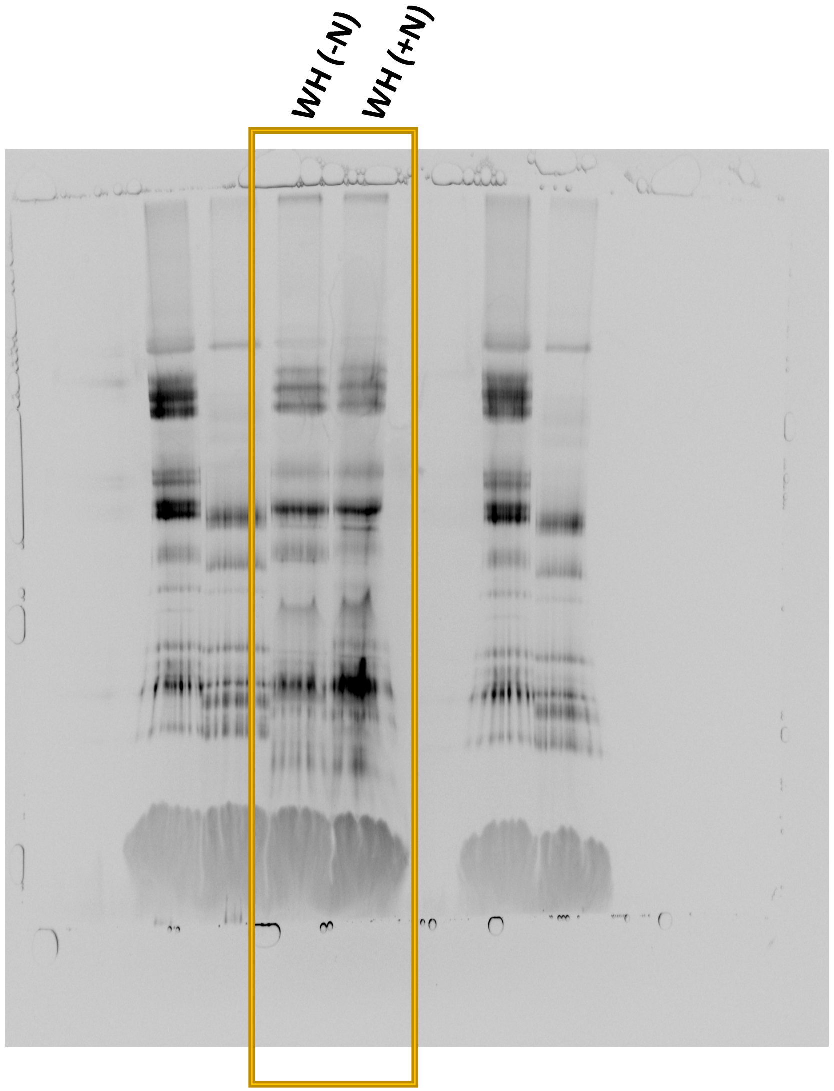

***Crocosphaera watsonii*_original uncropped gel images for Figure S2.** The center panel shows the original color scan (CN scan), and the right panel the original chlorophyll fluorescence scan (CN fluor). Blue and yellow boxes indicate the regions shown in the figure S2 (left).

Figure S2 | **CN scan** | **CN fluor**

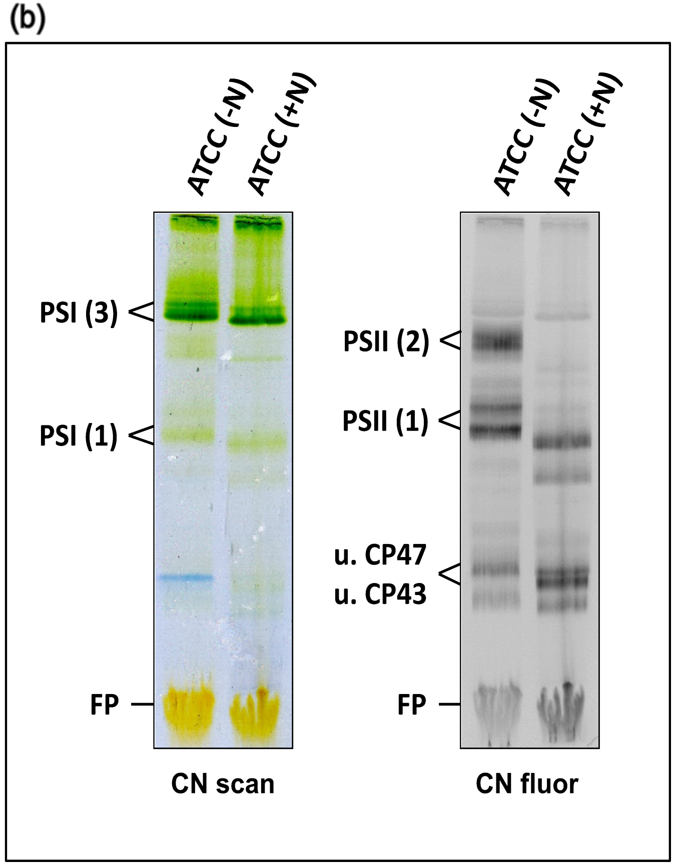

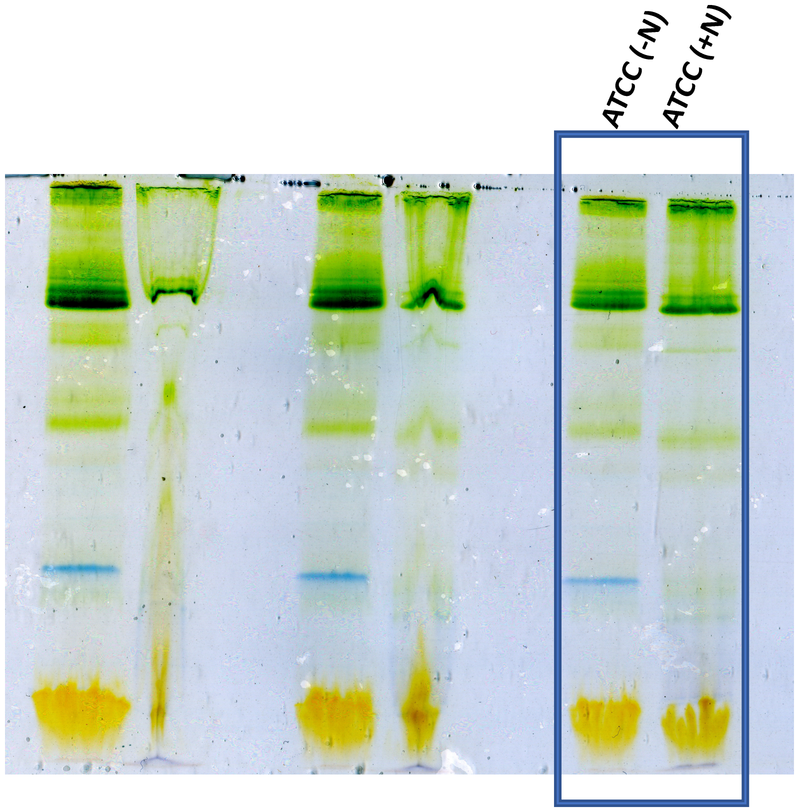

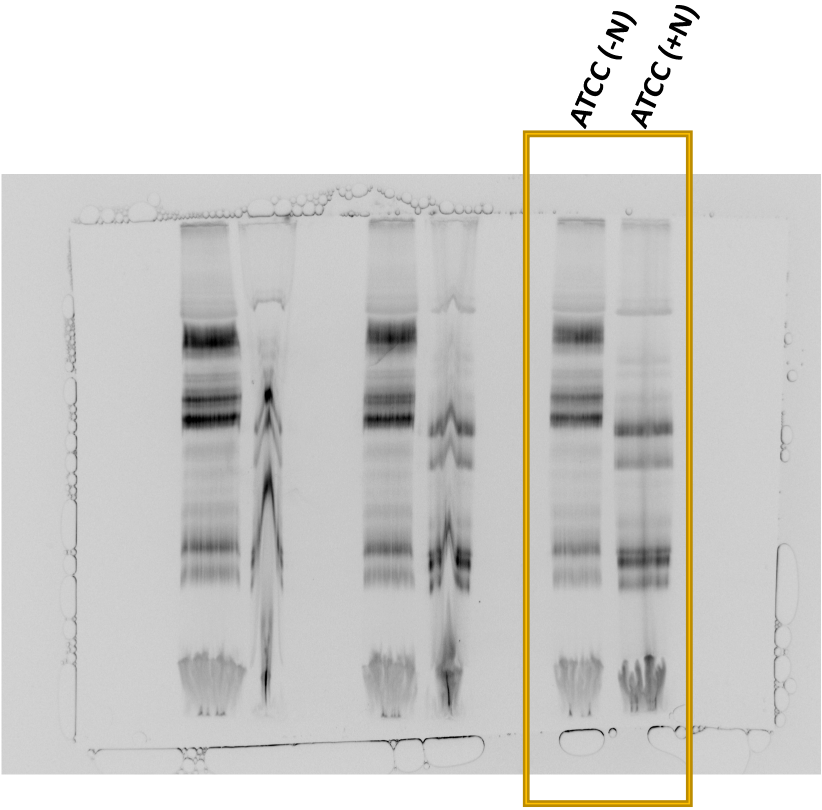

***Crocosphaera subtropica*_original uncropped gel images for Figure S2.** The center panel shows the original color scan (CN scan), and the right panel the original chlorophyll fluorescence scan (CN fluor). Blue and yellow boxes indicate the regions shown in the figure S2 (left).
