# Supplementary material for: Diazotrophy affects the state transitions in unicellular nitrogen fixing cyanobacteria
Source: Photosynth Res. 2026 Jul 27;164(4):43. doi: 10.1007/s11120-026-01231-3 (PMC13408156; doi:10.1007/s11120-026-01231-3)
Supplement: Supplementary file 2 — Supplementary Material 2 [file 11120_2026_1231_MOESM2_ESM.docx]

**Supplementary Information**

**Diazotrophy Affects the State Transitions in Unicellular Nitrogen Fixing Cyanobacteria**

Saverio Rana^1,2,*^, Tatsuhiro Tsurumaki^1^, Eva Kotabová^1^, Radek Kaňa^1,2^, Alžběta Prášilová^1^, Takako Masuda^3^, Ondřej Prášil^1,2,*^

^1^ Laboratory of Photosynthesis, Institute of Microbiology of the Czech Academy of Sciences, Centre Algatech, Novohradská 237, 379 01 Třeboň, Czech Republic

^2^ Faculty of Science, University of South Bohemia in České Budějovice, Branišovská 1645/31a, 370 05 České Budějovice, Czech Republic

^3^ Fisheries Resources Institute, Japan Fisheries Research and Education Agency, Shinha­macho, Shiogama, Miyagi, Japan

*corresponding authors: Saverio Rana, [rana@alga.cz](mailto:rana@alga.cz); Ondřej Prášil, [prasil@alga.cz](mailto:prasil@alga.cz)


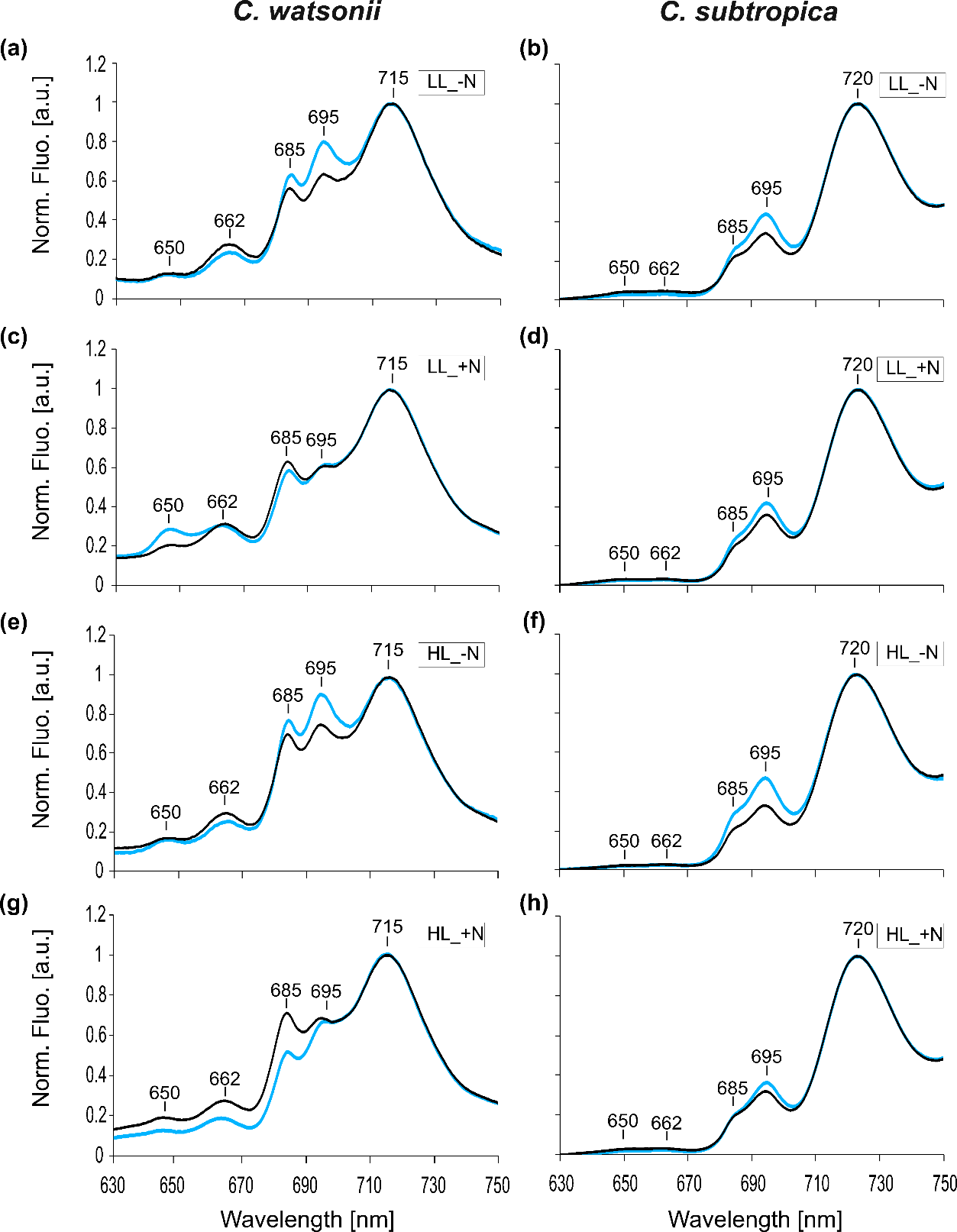


**Fig.S1 Low temperature (77 K) fluorescence emission spectra under Chl excitation.** 77 K fluorescence emission spectra (excitation at 450 nm) of *C*. *watsonii* (a, c, e, g) and *C*. *subtropica* (b, d, f, h) measured under diazotrophic (-N) and non-diazotrophic (+N) conditions, at low light (LL, 50 µmol photons m^-2^ s^-1^) and high light (HL, 200 µmol photons m^-2^ s^-1^). Blue lines represent samples measured 6 h into the light period (6L), whereas black lines represent samples collected at 6L and dark-acclimated for 10 min prior to measurement. Data represent averages from n=3 biological replicates. SD are not shown because of graphical reasons.

*****

*****


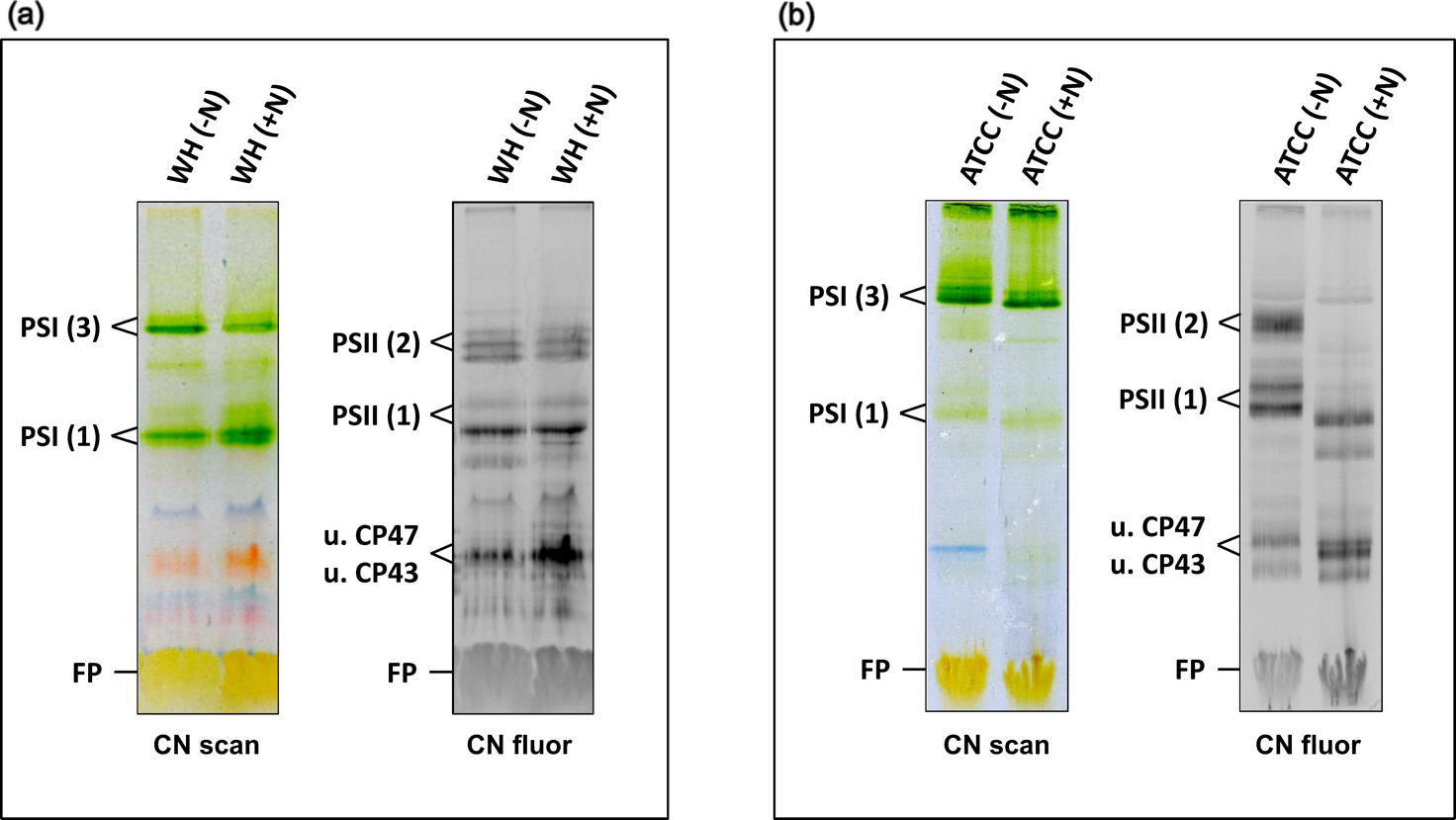


**Fig.S2** **Organization of photosystem complexes revealed by CN-PAGE.** Comparison of photosystem protein complexes by CN-PAGE. (a) *C. watsonii* and (b) *C. subtropica* under diazotrophic (-N) and non-diazotrophic (+N) conditions, grown at low light (LL, 50 µmol photons m^-2^ s^-1^). (a and b) the image of color scanning (Left: CN scan) and chlorophyll fluorescence image (Right: CN fluor). Diazotrophic cultures of *C. watsonii* showed higher PSI trimer abundance. Complex designations: PSI(3) and PSI(1), trimeric and monomeric photosystem I complexes, respectively; PSII(2) and PSII(1), dimeric and monomeric photosystem II complexes, respectively; u.CP47 and u.CP43, unassembled CP47 and CP43 proteins; FP, free pigments according to the references (Masuda et al. 2018; Masuda et al. 2022).


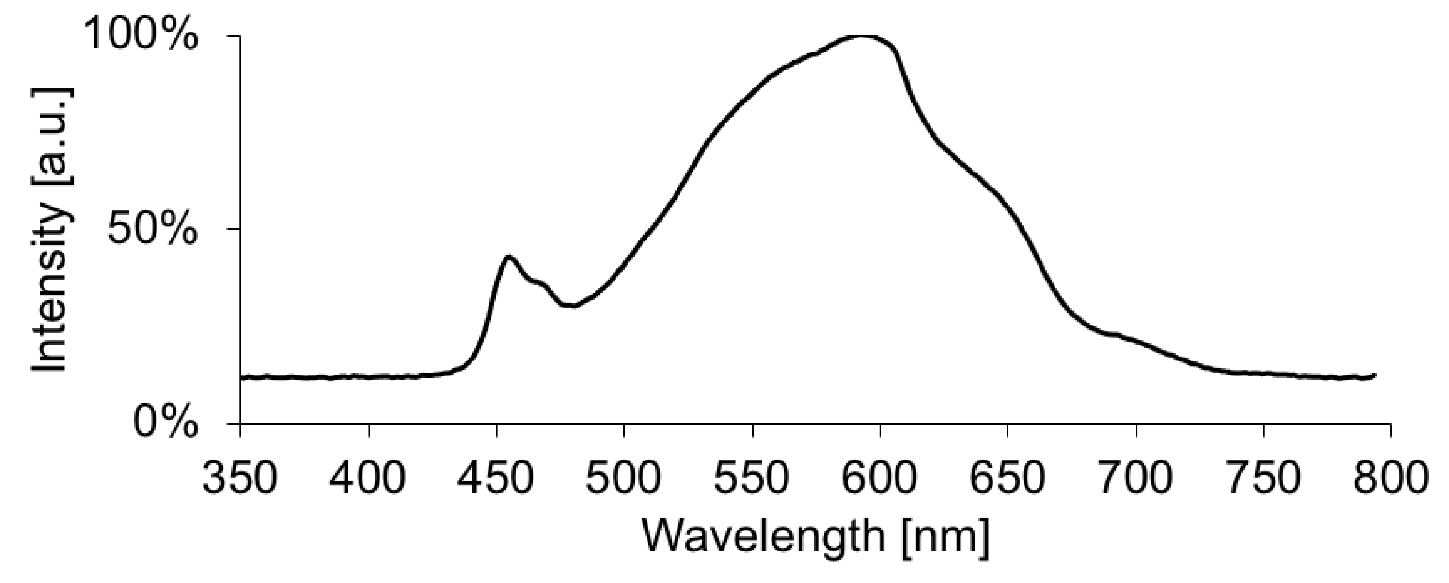


**Fig.S3 Emission spectrum of the warm white LED light source used in the MC1000 system.** The spectrum was measured in-house and is consistent with the manufacturer’s specification. The light source provides broad-spectrum illumination with characteristic contributions in the blue and red regions.
