# Supplementary material for: Diazotrophy affects the state transitions in unicellular nitrogen fixing cyanobacteria
Source: Photosynth Res. 2026 Jul 27;164(4):43. doi: 10.1007/s11120-026-01231-3 (PMC13408156; doi:10.1007/s11120-026-01231-3)
Supplement: Supplementary file 3 — Supplementary Material 3 [file 11120_2026_1231_MOESM3_ESM.docx]

**Diazotrophy Affects the State Transitions in Unicellular Nitrogen Fixing Cyanobacteria**

Saverio Rana^1,2,*^, Tatsuhiro Tsurumaki^1^, Eva Kotabová^1^, Radek Kaňa^1,2^, Alžběta Prášilová^1^, Takako Masuda^3^, Ondřej Prášil^1,2,*^

**Nitrogen-focused state transition statistical analysis**

*Methods and results summary for PBS/PSII, PSI/PSII, and capacity measurements*

# Summary

- **Main State transition capacity result:** State transition capacity was significantly higher under -N than +N in ATCC and WH at both 50 and 200 uE; all four planned contrasts remained significant after Holm correction.
- **State transition capacity ANOVA:** The Nitrogen main effect was very strong (p = 1.18e-09), with significant Species:Nitrogen and Species:LightIntensity:Nitrogen interactions.
- **Main PBS/PSII and PSI/PSII** **ratio result:** The clearest ratio result was WH PSI/PSII at 200 uE: +N was higher than -N using culture-level means across Light/Dark (Holm p across 8 tests = 0.0075).
- **Borderline ratio result:** WH PSI/PSII at 50 uE showed the same direction in culture-level means (raw p = 0.0083; Holm across all 8 tests = 0.058).
- **Light state-specific ratio result:** Direct comparisons showed WH PSI/PSII in Light at 200 uE was significantly higher under +N than -N after Holm correction across 16 tests.
- **Light state-response result:** Nitrogen changed the Light-Dark response for PSI/PSII, especially in WH and in ATCC at 200 uE.
- **Interpretation:** PBS/PSII showed no robust nitrogen effect after multiple-testing correction; the nitrogen signal is concentrated in State transition capacity and in the PSI/PSII ratio.

# Methods

## PBS/PSII and PSI/PSII ratio analyses

Three complementary analyses were performed for PBS/PSII and PSI/PSII. First, state-specific nitrogen tests compared -N with +N within the same strain, ratio, light/dark state, and light intensity. Second, because Light and Dark were paired repeated measurements of the same culture, a culture-level analysis averaged Light and Dark values for each replicate before testing -N vs +N. This culture-level analysis compared means at 50 uE and means at 200 uE. Third, Light-Dark responses were calculated for each culture as Light minus Dark, and these responses were compared between -N and +N to test whether nitrogen affected the magnitude or direction of the state response.

For pairwise tests of -N vs +N, Welch independent-samples t-tests were used because -N and +N were distinct treatment groups and equal variance was not assumed. Exact two-sided permutation tests were also calculated as robustness checks. Effect sizes were reported as Hedges g. P-values were adjusted for multiple testing with the Holm method and the Benjamini-Hochberg false discovery rate. Results were considered significant when the Holm-adjusted p-value was below 0.05.

## State transition capacity analyses

Capacity was analyzed with the same planned -N vs +N comparisons within each species and light intensity. In addition, a balanced 2 x 2 x 2 factorial ANOVA was computed with Species, Light Intensity, Nitrogen, and all interactions as fixed effects. The primary ANOVA term was Nitrogen. Interactions involving Nitrogen were used to evaluate whether the nitrogen effect differed between species and/or light intensities. Model diagnostics included Shapiro-Wilk residual normality, Brown-Forsythe/Levene variance homogeneity across groups, and a Kruskal-Wallis omnibus check across all Capacity groups.

# Results

## State transition capacity: planned -N vs +N comparisons

Capacity showed the clearest nitrogen effect. Capacity was higher under -N than +N for both species at both light intensities, and all four planned comparisons remained significant after Holm correction.

**Table 1. State transition capacity comparisons (-N vs +N).**

| **Species** | **Light (uE)** | **-N mean** | **+N mean** | **Difference (-N - +N)** | **95% CI** | **Raw p** | **Holm p** | **Direction** | **Signif.** |
| --- | --- | --- | --- | --- | --- | --- | --- | --- | --- |
| ATCC | 50 | 0.1369 | 0.0852 | 0.0517 | 0.0171 to 0.0862 | 0.0142 | 0.0142 | -N higher | * |
| ATCC | 200 | 0.1900 | 0.0784 | 0.1115 | 0.0562 to 0.1669 | 0.0051 | 0.0102 | -N higher | * |
| WH | 50 | 0.1671 | -0.0775 | 0.2446 | 0.1487 to 0.3406 | 0.0022 | 0.0087 | -N higher | ** |
| WH | 200 | 0.1146 | -0.0361 | 0.1508 | 0.0964 to 0.2051 | 0.0033 | 0.0099 | -N higher | ** |


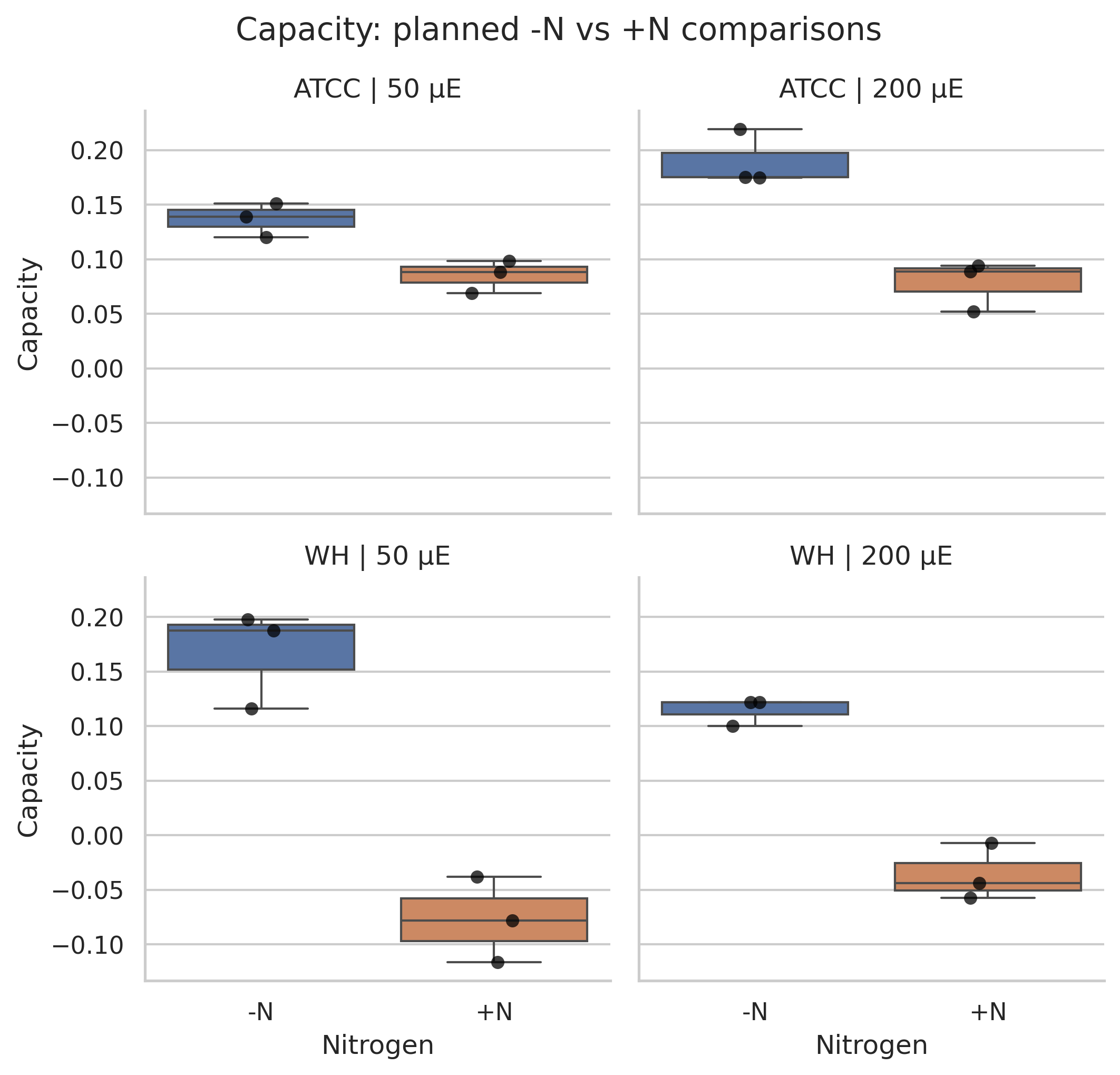


*Figure 1. Capacity values under -N and +N, faceted by species and light intensity.*


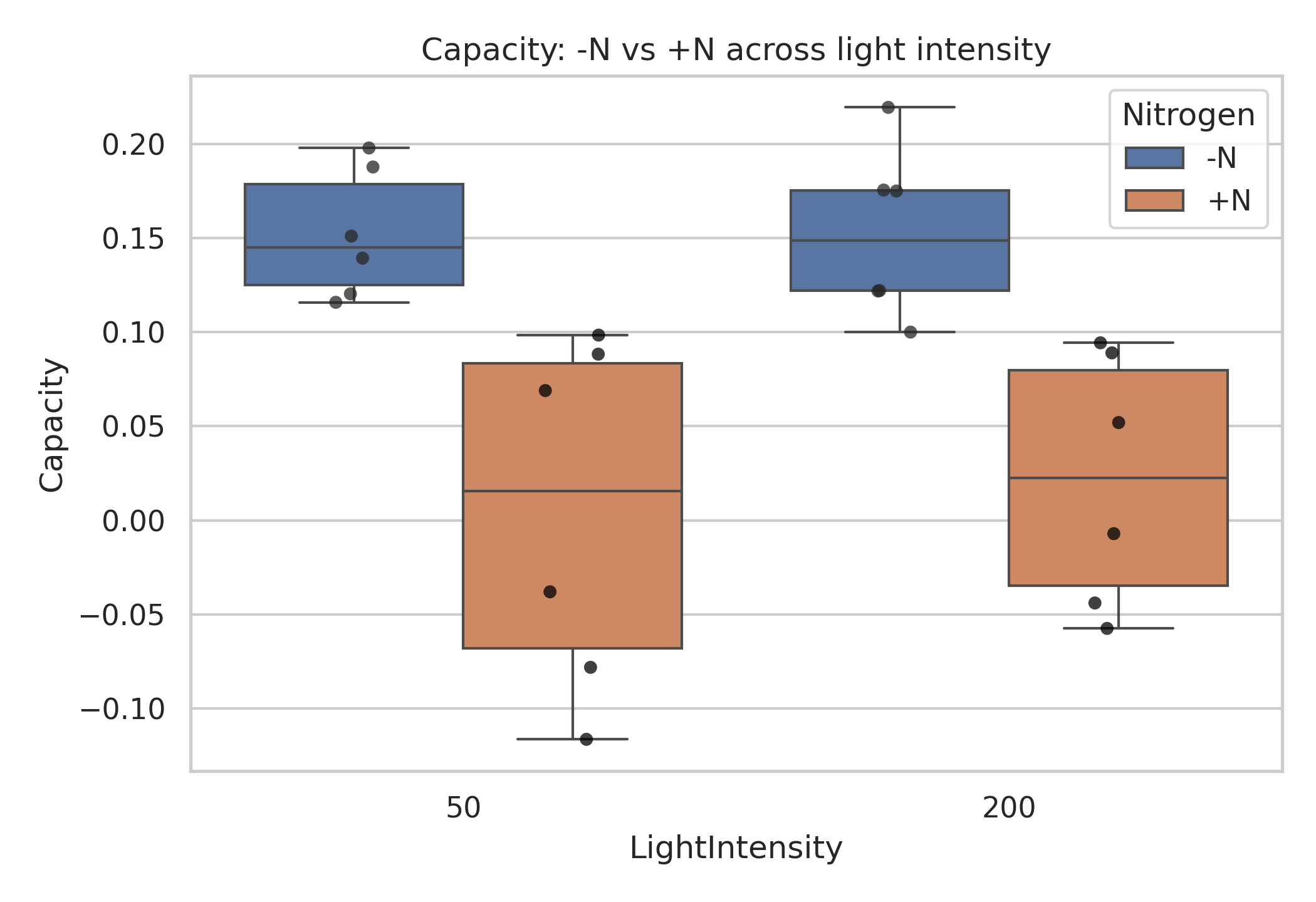


*Figure 2. Capacity values summarized by nitrogen condition and light intensity.*

## State transition capacity: factorial ANOVA

The factorial ANOVA confirmed a very strong nitrogen effect (p = 1.18e-09, partial eta-squared = 0.907). Species was also significant, and significant Species:Nitrogen and Species:Light Intensity:Nitrogen interactions indicated that the nitrogen response differed between strains and depended partly on light intensity. There was no significant overall Light Intensity main effect.

**Table 2. Capacity factorial ANOVA.**

| **Term** | **df** | **F** | **p** | **partial eta^2** |
| --- | --- | --- | --- | --- |
| Species | 1 | 51.759 | 2.14e-06 | 0.764 |
| LightIntensity | 1 | 0.614 | 0.4447 | 0.037 |
| Nitrogen | 1 | 155.463 | 1.18e-09 | 0.907 |
| Species:LightIntensity | 1 | 1.643 | 0.2182 | 0.093 |
| Species:Nitrogen | 1 | 26.861 | 9.07e-05 | 0.627 |
| LightIntensity:Nitrogen | 1 | 0.575 | 0.4594 | 0.035 |
| Species:LightIntensity:Nitrogen | 1 | 11.773 | 0.0034 | 0.424 |
| Residual | 16 |  |  |  |

## PBS/PSII and PSI/PSII ratio results: culture-level nitrogen analysis

The culture-level ratio analysis averaged Light and Dark values for each culture before testing nitrogen. The strongest result was WH PSI/PSII at 200 uE, where +N was higher than -N and remained significant after Holm correction across all eight culture-level ratio tests. WH PSI/PSII at 50 uE showed the same direction and was significant within the WH PSI/PSII block but borderline across all eight culture-level tests.

**Table 3. Culture-level ratio comparisons (-N vs +N after averaging Light/Dark).**

| **Species** | **Ratio** | **Light (uE)** | **-N mean** | **+N mean** | **Difference (-N - +N)** | **Raw p** | **Holm p (all 8)** | **Holm p (within ratio)** | **Direction** | **Signif. all 8** |
| --- | --- | --- | --- | --- | --- | --- | --- | --- | --- | --- |
| WH | PBS/PSII | 50 | 1.987 | 2.745 | -0.758 | 0.0713 | 0.3563 | 0.0837 | +N higher | ns |
| WH | PBS/PSII | 200 | 1.903 | 2.165 | -0.262 | 0.0418 | 0.2510 | 0.0837 | +N higher | ns |
| WH | PSI/PSII | 50 | 0.657 | 0.779 | -0.122 | 0.0083 | 0.0582 | 0.0083 | +N higher | ns |
| WH | PSI/PSII | 200 | 0.585 | 0.697 | -0.113 | 9.37e-04 | 0.0075 | 0.0019 | +N higher | ** |
| ATCC | PBS/PSII | 50 | 1.436 | 1.157 | 0.279 | 0.1063 | 0.4254 | 0.2127 | -N higher | ns |
| ATCC | PBS/PSII | 200 | 1.068 | 1.004 | 0.064 | 0.5278 | 1.0000 | 0.5278 | -N higher | ns |
| ATCC | PSI/PSII | 50 | 0.555 | 0.549 | 0.006 | 0.7783 | 1.0000 | 1.0000 | -N higher | ns |
| ATCC | PSI/PSII | 200 | 0.508 | 0.514 | -0.006 | 0.5807 | 1.0000 | 1.0000 | +N higher | ns |


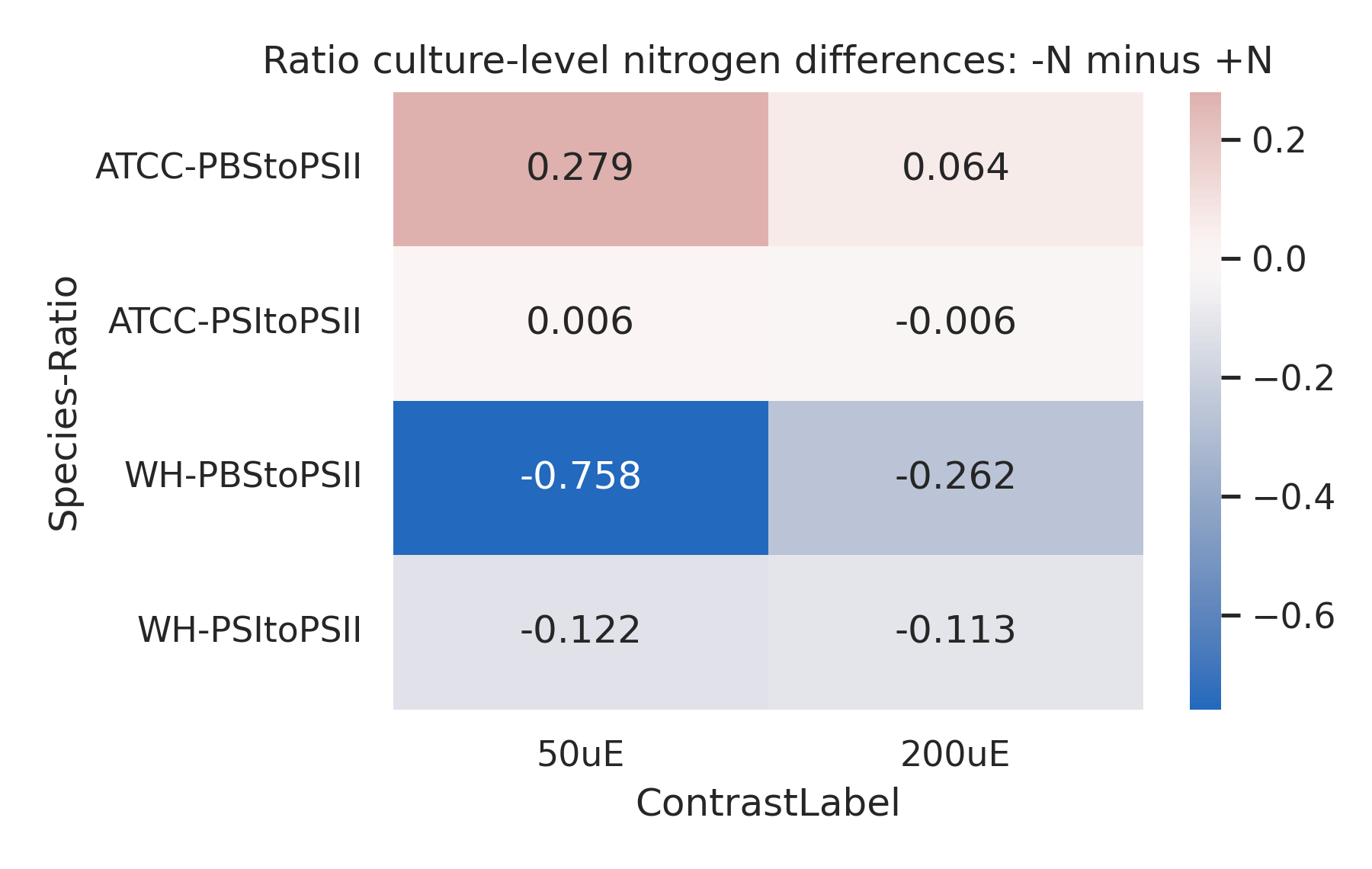


*Figure 3. Culture-level nitrogen differences for ratio data. Values are -N mean minus +N mean.*


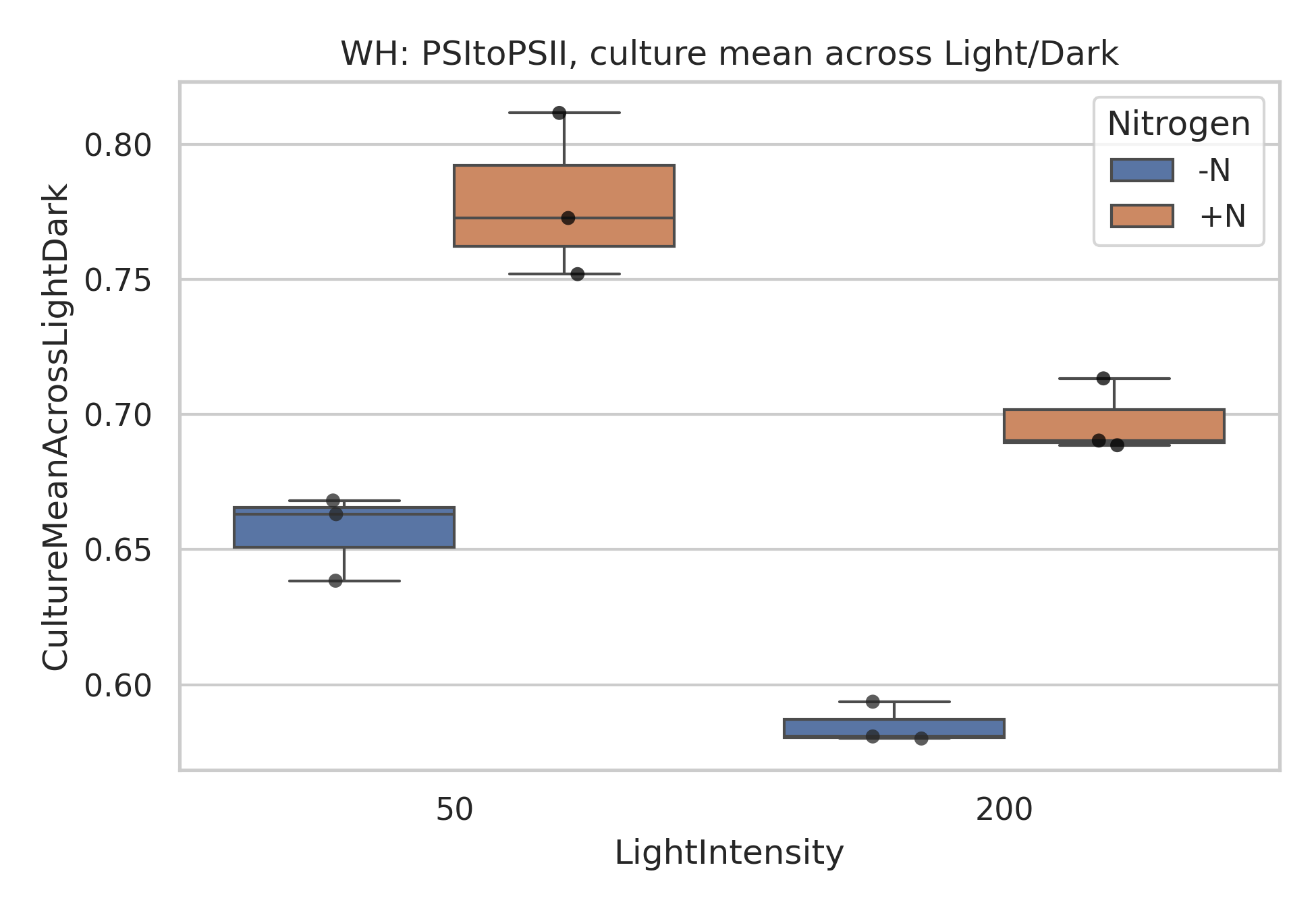


*Figure 4. WH PSI/PSII culture-level nitrogen comparison.*

## PBS/PSII and PSI/PSII Ratio results: light-specific comparisons

The light-specific analysis localized the nitrogen effect to particular Light/Dark states. After Holm correction across all 16 state-specific tests, the significant result was WH PSI/PSII in Light at 200 uE, where +N was higher than -N. PBS/PSII did not show a robust significant nitrogen effect after multiple-testing correction.

**Table 4. State-specific nitrogen comparisons for ratio data.**

| **Species** | **Ratio** | **State** | **Light (uE)** | **-N mean** | **+N mean** | **Difference (-N - +N)** | **Raw p** | **Holm p (all 16)** | **Holm p (within ratio)** | **Direction** | **Signif. all 16** |
| --- | --- | --- | --- | --- | --- | --- | --- | --- | --- | --- | --- |
| WH | PBS/PSII | Light | 50 | 1.875 | 3.595 | -1.720 | 0.0445 | 0.4900 | 0.1782 | +N higher | ns |
| WH | PBS/PSII | Light | 200 | 1.440 | 1.853 | -0.413 | 0.0526 | 0.5264 | 0.1782 | +N higher | ns |
| WH | PBS/PSII | Dark | 50 | 2.099 | 1.895 | 0.204 | 0.4116 | 1.0000 | 0.7186 | -N higher | ns |
| WH | PBS/PSII | Dark | 200 | 2.365 | 2.477 | -0.112 | 0.3593 | 1.0000 | 0.7186 | +N higher | ns |
| WH | PSI/PSII | Light | 50 | 0.573 | 0.818 | -0.245 | 0.0082 | 0.1235 | 0.0247 | +N higher | ns |
| WH | PSI/PSII | Light | 200 | 0.528 | 0.716 | -0.188 | 0.0011 | 0.0169 | 0.0042 | +N higher | * |
| WH | PSI/PSII | Dark | 50 | 0.740 | 0.740 | 0.000 | 0.9964 | 1.0000 | 0.9964 | -N higher | ns |
| WH | PSI/PSII | Dark | 200 | 0.642 | 0.679 | -0.037 | 0.0268 | 0.3211 | 0.0535 | +N higher | ns |
| ATCC | PBS/PSII | Light | 50 | 1.055 | 1.075 | -0.020 | 0.7946 | 1.0000 | 0.7946 | +N higher | ns |
| ATCC | PBS/PSII | Light | 200 | 0.787 | 0.869 | -0.081 | 0.0964 | 0.7805 | 0.3469 | +N higher | ns |
| ATCC | PBS/PSII | Dark | 50 | 1.817 | 1.239 | 0.578 | 0.0867 | 0.7805 | 0.3469 | -N higher | ns |
| ATCC | PBS/PSII | Dark | 200 | 1.349 | 1.139 | 0.209 | 0.3498 | 1.0000 | 0.6997 | -N higher | ns |
| ATCC | PSI/PSII | Light | 50 | 0.486 | 0.507 | -0.020 | 0.3673 | 1.0000 | 0.4174 | +N higher | ns |
| ATCC | PSI/PSII | Light | 200 | 0.413 | 0.474 | -0.062 | 0.0239 | 0.3105 | 0.0716 | +N higher | ns |
| ATCC | PSI/PSII | Dark | 50 | 0.623 | 0.592 | 0.031 | 0.2087 | 1.0000 | 0.4174 | -N higher | ns |
| ATCC | PSI/PSII | Dark | 200 | 0.603 | 0.553 | 0.050 | 0.0149 | 0.2091 | 0.0598 | -N higher | ns |


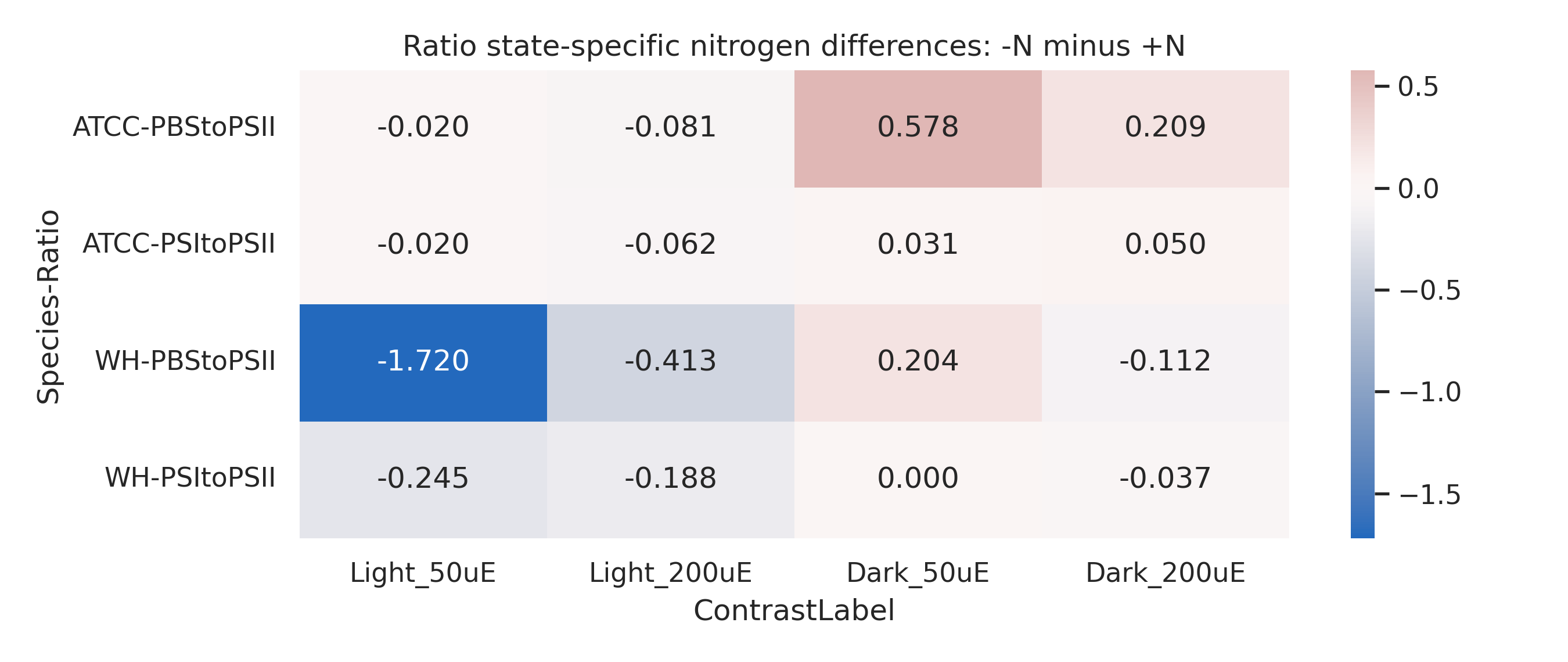


*Figure 5. Light state-specific nitrogen differences for ratio data. Values are -N mean minus +N mean.*


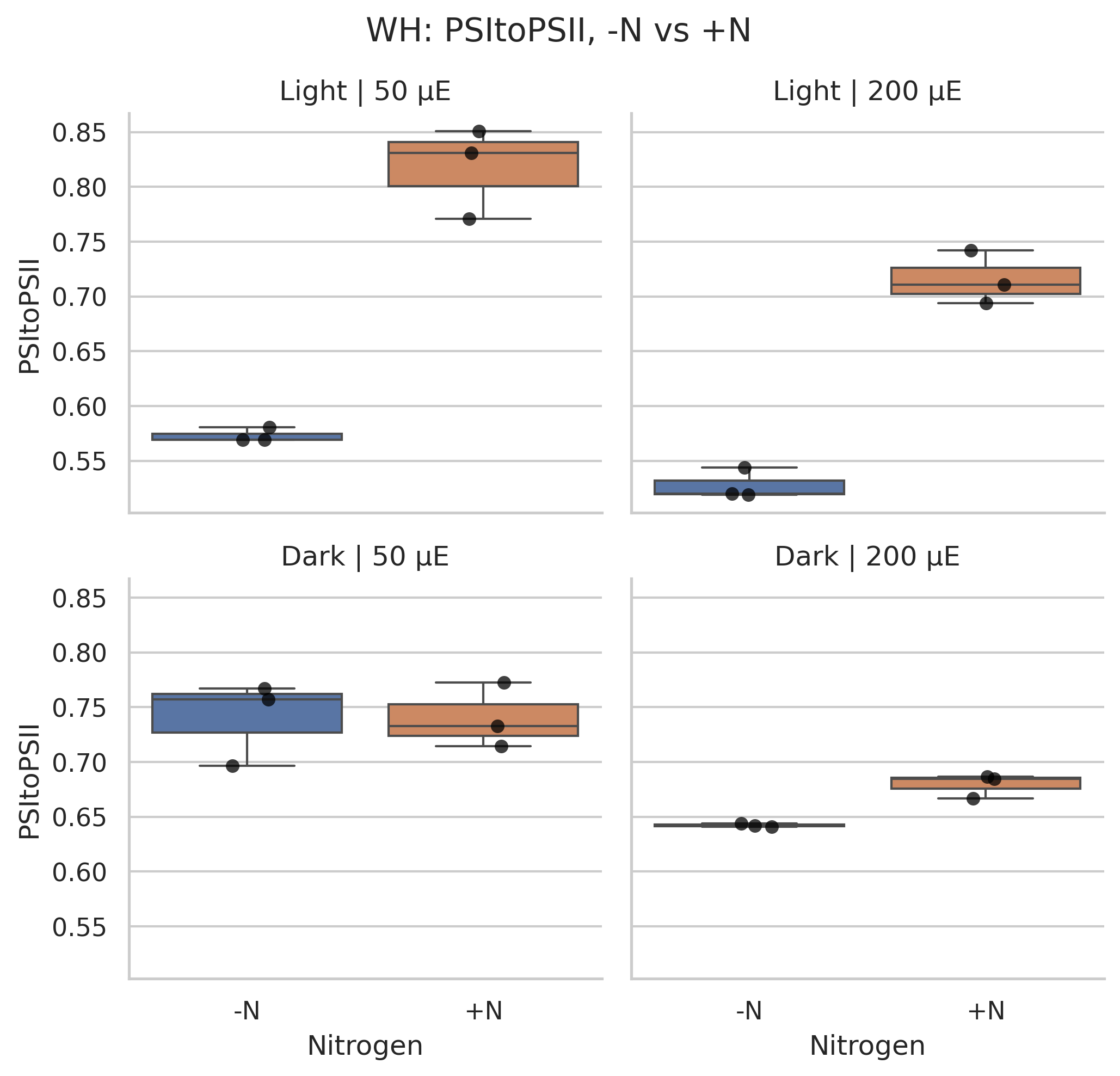


*Figure 6. WH PSI/PSII light state-specific nitrogen comparisons.*

## Nitrogen effect on the Light-Dark response

Because Light and Dark were paired within culture, Light-Dark responses were calculated as Light minus Dark. Nitrogen significantly changed the Light-Dark response for WH PSI/PSII at both light intensities and for ATCC PSI/PSII at 200 uE after Holm correction across eight tests. This indicates that nitrogen affects not only absolute PSI/PSII values, but also the magnitude and/or sign of the Light-Dark response.

**Table 5. Nitrogen effect on Light-Dark responses.**

| **Species** | **Ratio** | **Light (uE)** | **-N Light-Dark response** | **+N Light-Dark response** | **Difference** | **Raw p** | **Holm p (all 8)** | **Signif.** |
| --- | --- | --- | --- | --- | --- | --- | --- | --- |
| WH | PBS/PSII | 50 | -0.224 | 1.700 | -1.924 | 0.0311 | 0.1243 | ns |
| WH | PBS/PSII | 200 | -0.924 | -0.624 | -0.300 | 0.1688 | 0.3375 | ns |
| WH | PSI/PSII | 50 | -0.167 | 0.077 | -0.245 | 0.0022 | 0.0175 | * |
| WH | PSI/PSII | 200 | -0.115 | 0.036 | -0.151 | 0.0033 | 0.0232 | * |
| ATCC | PBS/PSII | 50 | -0.762 | -0.164 | -0.598 | 0.0788 | 0.2365 | ns |
| ATCC | PBS/PSII | 200 | -0.561 | -0.270 | -0.291 | 0.2416 | 0.3375 | ns |
| ATCC | PSI/PSII | 50 | -0.137 | -0.085 | -0.052 | 0.0142 | 0.0711 | ns |
| ATCC | PSI/PSII | 200 | -0.190 | -0.078 | -0.112 | 0.0051 | 0.0305 | * |

## Diagnostics and robustness checks

Capacity model diagnostics did not indicate major violations: Shapiro-Wilk residual normality p = 0.815, Brown-Forsythe/Levene variance p = 0.918, and Kruskal-Wallis across A-H p = 0.00393. Exact permutation p-values were calculated for pairwise tests, but with n = 3 per group the possible permutation p-values are coarse and should be treated as sensitivity checks rather than the main criterion.

**Table 6. Capacity diagnostics.**

| **Diagnostic** | **Statistic** | **p** |
| --- | --- | --- |
| Shapiro-Wilk residual normality | 0.976 | 0.8146 |
| Levene/Brown-Forsythe variance | 0.350 | 0.9182 |
| Kruskal-Wallis A-H | 20.893 | 0.0039 |

# Interpretation

The most robust nitrogen-dependent phenotype is State transition capacity: -N consistently increased capacity relative to +N across both strains and light intensities. The factorial ANOVA indicates that this nitrogen effect is large, but it is not identical across species and light contexts. In the PBS/PSII and PSI/PSII ratio data, the nitrogen signal is more specific: the most reproducible effect is in PSI/PSII, especially in WH at 200 uE, where +N increased PSI/PSII relative to -N. The PBS/PSII ratio did not show a statistically robust nitrogen effect after multiple-testing correction.
